# Gut microbiota: a new factor modulating the immunizing potential of viral and cancer vaccines

**DOI:** 10.21203/rs.3.rs-4294379/v1

**Published:** 2025-10-28

**Authors:** Agathe Carrier, Paolo Manghi, Carolina Alves Costa Silva, Imran Lahmar, Deborah Suissa, Valerio Iebba, Andrew A. Almonte, Nadim J. Ajami, Roxanne Birebent, Caroline Laheurte, Gerty Schreibelt, Louis Lemant, Jean-Eudes Fahrner, Eric de Sousa, Franck Berthier, Juliette Villemonteix, Mathieu F Chevalier, Gianmarco Piccinno, Didier Hocquet, Isabelle Lebhar, Markus Maeurer, Sophie Caillat-Zucman, Sebastian Kobold, Guido Kroemer, Lisa Derosa, Odile Launay, Dietmar H. Pieper, Marius Vital, Antonio Santos-Peral, Magdalena Zaucha, María García-Bengoa, Julia Thorn-Seshold, Helen Stirling, Encouse Golden, Kalijn F. Bol, Michael G. White, Pranoti V. Sahasrabhojane, Yasmine M. Hoballah, Jillian S. Losh, Carolyn R. DePinho, Eleonora Dondossola, I. Jolanda M. de Vries, Olivier Adotevi, Jennifer A. Wargo, Simon Rothenfußer, Nicola Segata, Silvia Formenti, Laurence Zitvogel

**Affiliations:** 1. Université Paris-Saclay, Faculté de Médecine, Le Kremlin-Bicêtre, France.; 2. Gustave Roussy Cancer Campus (GRCC), Clinicobiome, 94800 Villejuif Cedex, France.; 3. Institut National de la Santé Et de la Recherche Médicale (INSERM) U1015, Equipe Labellisée-Ligue Nationale contre le Cancer, 94800 Villejuif, France.; 4. Department CIBIO, University of Trento, Trento, Italy.; 5. Computational Biology Unit, Research and Innovation Centre, Fondazione Edmund Mach, via Mach 1, 38098 San Michele all’Adige, Italy; 6. Platform for Innovative Microbiome and Translational Research, The University of Texas MD Anderson Cancer Center, Houston, TX, USA.; 7. Department of Genomic Medicine, The University of Texas MD Anderson Cancer Center, Houston, TX, USA.; 8. Université de Franche-Comté, EFS, INSERM, UMR 1098 RIGHT, F-25000 Besançon, France.; 9. Medical BioSciences, Radboud university medical center, Nijmegen, the Netherlands.; 10. ImmunoTherapy/ImmunoSurgery, Champalimaud Centre for the Unknown, Lisboa, Portugal.; 11. Life Sciences, R&D BioMérieux, Marcy l’Etoile, France.; 12. Laboratoire d’Immunologie et Histocompatibilité, Hôpital Saint-Louis, AP-HP, Université de Paris, 75010 Paris, France.; 13. INSERM UMR 976, Institut de Recherche Saint-Louis, Université Paris Cité, Paris, France.; 14. Université de Franche-Comté, UMR 6249 Chrono-environnement, F-25000 Besançon, France; CHU de Besançon, Hygiène Hospitalière, F-25000 Besançon, France.; 15. Medizinische Klinik, Johannes Gutenberg University Mainz, Germany.; 16. Division of Clinical Pharmacology, LMU University Hospital, Munich, LMU Munich, 80336 Munich, Germany.; 17. German Cancer Consortium (DKTK), a partnership between LMU University Hospital and DKFZ, Heidelberg, Germany.; 18. Einheit für Klinische Pharmakologie (EKLiP), Helmholtz Zentrum München-German Research Center for Environmental Health Neuherberg, 85764 Oberschleißheim, Germany.; 19. Centre de Recherche des Cordeliers, INSERM U1138, Équipe Labellisée - Ligue Nationale contre le Cancer, Université Paris Cité, Sorbonne Université, Paris, France.; 20. Metabolomics and Cell Biology Platforms, Gustave Roussy Cancer Campus, Villejuif, France.; 21. Institut du Cancer Paris CARPEM, Department of Biology, Hôpital Européen Georges Pompidou, AP-HP, Paris, France.; 22. Université Paris Cité; Inserm CIC 1417, I-Reivac; APHP, Hôpital Cochin, Paris, France.; 23. Microbial Interactions and Processes Research Group, Helmholtz Centre for Infection Research, 38124 Braunschweig Germany.; 24. Institute of Medical Microbiology and Hospital Epidemiology, Hannover Medical School, Hannover, Germany.; 25. Faculty of Chemistry and Pharmacy, LMU Munich, Munich, Germany.; 26. Department of Radiation Oncology, Weill Cornell Medicine, New York.; 27. Department of Medical Oncology, Radboud university medical center, Nijmegen, the Netherlands.; 28. Department of Colon & Rectal Surgery, The University of Texas MD Anderson Cancer Center, Houston, TX, USA.; 29. Platform for Innovative Microbiome and Translational Research, The University of Texas MD Anderson Cancer Center, Houston, TX, USA.; 30. Department of Genitourinary Medical Oncology, The University of Texas MD Anderson Cancer Center, Houston, TX, USA.; 31. Department of Medical Oncology, CHU, Besançon, France.; 32. Department of Surgical Oncology, The University of Texas MD Anderson Cancer Center, Houston, TX, USA; 33. Department of Experimental Oncology, IEO European Institute of Oncology IRCCS, Milan, Italy.; 34. Sandra and Edward Meyer Cancer Center, New York, NY, USA.; 35. Department of Medicine, Weill Cornell Medical College, New York, NY, USA.; 36. Center of Clinical Investigations in Biotherapies of Cancer (BIOTHERIS), Villejuif, France.

**Keywords:** Vaccines, Microbiota, COVID-19, cancer, T cell assays, Th1, Th2, Dendritic cells, peptides, MetaPhlAn 4

## Abstract

Vaccines represent a major public health intervention against infectious diseases and potentially cancer. Surrogate markers of vaccine efficacy usually rely on neutralizing antibody titers afflicted by high interindividual variabilities. Automated multiplexed T cell assays currently allow to test the clinical relevance of T lymphocyte responses during vaccine rollout. We examined cellular and/or humoral immune responses in five independent cohorts of health care workers, young healthy individuals and patients with cancer (melanoma or lung cancer) receiving various immunizing formulations (non-replicating, viral/tumoral, mRNA/peptides/cellular/viral particles). Here we show that about 20% of vaccinees to non-replicating formulations fail to mount protective antibody and Th1/Tc1 responses while 9% receiving a live vaccine were hyperresponders. Vaccine outliers could at least in part be attributed to gut dysbiosis at baseline, evaluated by shotgun metagenomics-based machine learning or the TOPOSCORE. These findings highlight the requirement of diagnostic tools to identify intestinal dysbiosis, as well as microbiota-centered interventions to optimize the efficiency of mass vaccinations.

## Introduction

Vaccines are considered as one of the most efficient primary public health interventions against severe infectious diseases, saving countless lives since the late eighteenth century^1^. Although antibody (Ab) titers have been used as surrogates of vaccine efficacy for many decades, high affinity neutralizing Ab produced by effector B cells cooperate with long lived memory T cell responses to mediate full-fledged clinical protection^2^. Vaccines against severe acute respiratory syndrome coronavirus 2 (SARS-CoV-2) were developed at an unprecedented speed to fight the coronavirus disease 2019 (COVID-19) pandemic. Randomized trials have shown that these vaccines dramatically reduced symptomatic COVID-19^3^. Nevertheless, we still face many challenges in the development of vaccines against major viruses, such as human immunodeficiency virus (HIV), hepatitis C virus and influenza virus, in part due to intrinsic features related to the virus, the vaccine design and its route of administration, as well as host characteristics.

Regarding the latter parameters, there are broad interindividual variations in the magnitude and duration of immune responses monitored after vaccination campaigns. Hence, neutralizing antibody titers, hemagglutinin inhibition titers and pneumococcus-specific immunoglobulin (Ig) G concentrations could vary from 10 to 100-fold among vaccinees receiving live attenuated yellow fever (YF) 17D vaccine^4^, inactivated seasonal influenza vaccine^5^ or pneumococcal conjugate vaccine^6^, respectively. Similarly, Bacille de Calmette Guerin (BCG)-vaccinated infants displayed considerable variability in their cytokine or T cell responses to mycobacterial antigens as well as in the protection conferred against lung tuberculosis^7,8^. The capacity of the diphtheria-tetanus-pertussis (DTP) vaccine in conveying protection against mild symptoms only pertains to 70 to 80% of individuals^9^. Moreover, clinical trials of vaccine rollout in developing countries reported a lower immunogenicity than in the Western world-vaccinated infants^10,11^.

Given the importance of the intestinal microbiota in the regulation of systemic inflammation and immunity^12,13^, the impact of the gut commensalism on the immunization efficiency of vaccines has been analyzed in experimental mouse models and epidemiological studies^14–16^. Pioneer studies concluded that antibiotics (ABX) compromise humoral immune responses in association with ABX-induced dysbiosis^17^. Not only ABX but also chronic inflammatory disorders including cancer can induce a state of “gut dysbiosis” characterized by the relative over dominance of the genus *Enterocloster*, oral taxa and pathobionts at the relative expense of health-associated commensals (such as other *Lachnospiraceae* and *Oscillospiraceae* family members, as well as *Akkermansia muciniphila*)^18–20^. Recently, a custom scoring based on the ecological topology of intestinal microbiota, called TOPOSCORE has been reported to allow the evaluation of the balance between beneficial (SIG2, immunogenic) versus harmful (SIG1, tolerogenic) bacteria in the context of cancer patients treated with immune checkpoint inhibitors^21^.

There is no knowledge on the impact of the intestinal microbiota on vaccine-induced memory T cell responses and their clinical consequences. To fill this gap, we conducted a prospective study on 75 health care workers (HCW) who received SARS-CoV-2 vaccines during the first wave of the COVID-19 pandemic (Weill Cornell University, NYC, USA). We also analyzed two clinical trials in 57 patients with melanoma (NCT02993315, Netherlands)^22,23^, who were vaccinated with autologous dendritic cells (DC) pulsed with melanoma peptides, and 26 patients with lung cancer (NCT02818426, France)^24^, who were vaccinated with telomerase peptides. These three studies aimed at correlating shotgun metagenomics (MG)-based taxonomic composition of stools at baseline (pre-vaccination) with T cell responses to vaccine peptides and with RBD-S1-specific antibody titers in HCW. We observed that a healthy microbiome (characterized by *Lachnospiraceae* and *Oscillospiraceae* family members, such as *Faecalibacterium prausnitzii* and *Dorea longicatena*) was strongly associated with the elicitation of robust vaccine-specific polyfunctional Th1/Tc1 immune responses, while patients with high relative abundances of *Flavobacteriales, Proteobacteria* or *Eggerthellaceae*, which are typical of chronic inflammation-related dysbiosis and immunoresistance, exhibited T cell hyporeactivity. The TOPOSCORE calculation also attributed vaccine failure to a gut dysbiosis in all three cohorts. In another small cohort of 35 healthy US individuals, 40% did not exhibit a rise in Ab titers after the third SARS-CoV-2 vaccine booster, that coincided with a relative poorer fecal richness and more inflammatory stool composition.

In a fourth cohort of 174 young individuals vaccinated with the live-attenuated yellow fever 17D (YF17D) vaccine, a model of acute viral infection inducing long lasting immunity, the gut taxonomic composition was markedly associated with the 9% outliers developing the strongest vaccine response according to a composite score comprising neutralizing antibodies as well as vaccine-specific CD8 and CD4 T cell responses.

## Results

### Twenty-five percent of French HCW failed to mount protective RBD-S1-specific Th1/Tc1 immune responses across all waves of the COVID-19 pandemic.

We previously reported that the polarity and antigen specificity of preexisting T lymphocyte responses against SARS-CoV-2 determined susceptibility to SARS-CoV-2 *de novo* infection or breakthrough infection post-vaccination during the first wave of the COVID-19 pandemics^25^. Thus, in a large cohort (called “COV3APHP” and “ONCOVID”) of 368 HCW and patients with cancer, Th1/Tc1 (but not Th2) responses against receptor binding domain (RBD)-spike-1 (S1) stood out for protecting against SARS-CoV-2 viral infection between April and September 2020. We showed that two immunizations with the BNT162b2 mRNA vaccine (BioNTech/Pfizer) and/or AZD1222 adenovirus (AstraZeneca) triggered anti-RBD-S1 specific Th1/Tc1 responses in 49%, 34% and 10% of individuals without cancer, with solid tumor and with hematologic cancers, respectively^25^. Our previous work also concluded that Th1/Tc1 cell immunity against the RBD sequences of alpha and delta viral variants was drastically reduced compared with the reactivity towards wild type (WT) sequences in these individuals^25^.

In the current study, we longitudinally measured T cell responses directed against RBD-S1 WT sequence in 160 HCW of the COV3APHP cohort and 13 cancer patients within the ONCOVID^25^ cohort over time across vaccine boosts and COVID-19 viral infections until December 2022 (**Fig. S1A, Table S1**). We used the same methodology as previously described^25^, based on a simple 22 hour-whole blood stimulation assay allowing the quantitative measurement of interferon gamma (IFNγ) using an enzyme linked fluorescent assay (ELFA) in an automated platform (VIDAS^®^-30 IFNγ Research Use Only)^26^. We monitored RBD-S1-specific T cell reactivities against a pool of eleven non-overlapping peptides (“PEPwtRBD”) (**Table S2A**) to determine inter- and intra-individual variations in IFNγ release induced by vaccination or viral infection (**Fig. S1B**). Surprisingly, 25% of individuals failed to ever mount PEPwtRBD-specific Th1/Tc1 immune responses (**Fig. S1C**). Most of these individuals (82%) also failed to respond to 14 mutated RBD-S1 sequences of viral variants of concern (VOC) Alpha (B.1.1.7), Beta (B.1.351), Gamma (P.1) and Delta (B.1.617.2), henceforth called “PEPmutRBD” (**Table S2A; Fig. S1D**).

Altogether, despite a complete vaccination program and exposure to several SARSCoV-2 viral waves during the pandemic, 20% of healthy individuals failed to mount protective Th1/Tc1 immune responses against the WT and the mutated RBD-S1 sequences.

### No links between HLA genotypes and non-reactivity against RBD-S1.

Human leukocyte antigens (HLA) genotypes may account for disparities in the immune response to RBD-S1^27–29^. Accordingly, COVID-19 patients bearing certain HLA alleles (such as HLA-B*07:02, -C*07:02, -A*03:01) are more likely to possess coronavirus 2 human coronavirus (CoV-2-HCoV) cross-reactive CD8^+^ T cells^30^ that can be reamplified by vaccine shots. The binding affinity of RBD-S1 peptides to major histocompatibility complex (MHC) class I and class II proteins was calculated by means of the NetMHCpan algorithm. This approach predicted strong binding of the RBD epitopes “SPIKE25” (residues 361–375), “SPIKE27” (residues 391–405) and “SPIKE31” (residues 451–465) to MHC class I HLA-A, -B and -C alleles. In contrast, “SPIKE33” (residues 481–495) was estimated to have a low affinity for HLA-B and no affinity for HLA-C alleles (**Table S2b.1**). Only “SPIKE24”, “SPIKE25”, “SPIKE31” and “SPIKE35” exhibit a high affinity for MHC class II HLA-DR (**Table S2b.2**), as previously reported for the immunodominant S346–365 region^31^. To investigate potential links between the lack of spontaneous or vaccine-induced RBD-S1-specific Th1/Tc1 responses and HLA genotypes, we analyzed the distribution of HLA-I and HLA-II alleles in 59 individuals, including 46 HCW and 13 patients with cancer after vaccination, among whom 44% were tested more than twice in independent assays and 29% failed to mount RBD-S1 Th1/Tc1 responses (**Table S1a**). We found significant associations between specific HLA class I (such as HLA-A*32:01) and class II alleles and the absence of RBD-S1-specific Th1/Tc1 responses, with some commonalities with severe COVID-19^32^ (**Fig. S1E**). Next, we analyzed the distribution of HLA-I and HLA-II alleles in 114 individuals of a new cohort “COV3APHP” of HCW drawn after the vaccine booster and several SARS-CoV-2 viral waves, among whom 77% were tested more than 4 times in independent assays and 25% failed to mount RBD-S1-specific Th1/Tc1 responses (**Table S1b**). Here, we found two HLA alleles that were positively associated with vaccine responses (HLA-DQB1*06:02, HLA-DRB1*15:01), corroborating a prior study^33^, as well as four HLA alleles (HLA-B*08:01, HLA-C*07:02, HLA-A*01:01, HLA-DRB1*13:02) associated with absent T cell responses against RBD-S1 (**Fig. S1F)**. Augusto et al. also identified a strong association between HLA-B*15:01 and asymptomatic infections in two independent cohorts^34^.

Since there was no commonality between the two cohorts with respect to HLA alleles associated to failed Th1/Tc1 responses against RBD-S1 (**Fig. S1E-F)**, we conclude that, in these small cohorts, there was no strong evidence that absent vaccine responses would be associated with HLA.

### 25% of American HCW failed to mount protective RBD-S1-specific Th1/Tc1 immune responses after the first round of COVID-19 vaccines (WELCOME study).

**Figure 1.**
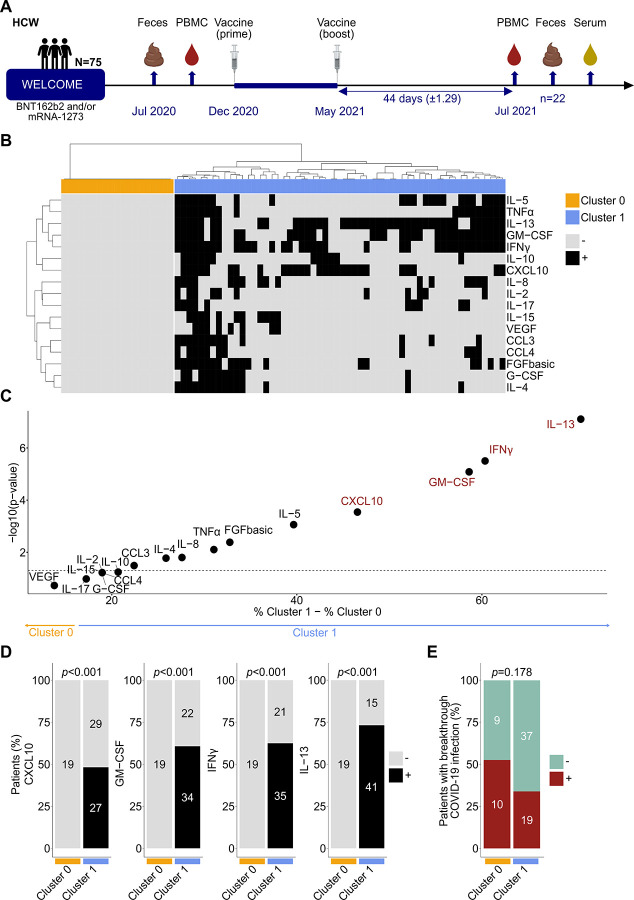
Failure of COVID-19 vaccines to elicit RBD-S1-specific Th1/Tc1 immune responses. **A.** Graphical representation of the WELCOME patient cohort’s protocol with fecal and blood collection and timelines between vaccination and sampling (refer to **Table S3**). **B.** Bicolor map of cytokine secretion after 72h PBMC stimulation with PEPwtRBD peptide pool (positive in black, negative in grey). Patients (n=75) were ordered in columns and cytokines were ordered in rows by unsupervised hierarchical clustering using ward.D2 clustering method and binary distance calculation method. Cytokines with less than 10% frequency were excluded of this clustering. Heatmap was cut in 2 clusters of patients (orange: non-reactive (cluster 0), light blue: polyreactive Th1/Tc1 (cluster 1)). **C.** Volcano plot depicting the most significant cytokines after PBMC stimulation with the PEPwtRBD peptide pool between cluster 1 (light blue) and cluster 0 (orange) of panel B. Volcano plot was generated computing delta of the percentages of positive patients in cluster 1 and the percentages of positive patients in cluster 0 for each cytokine (x axis) with the −log10 of the p-values deriving from Fisher’s exact test for each cytokine (y axis). **D.** Barplots representing the percentage and number of positive patients (black) in each cluster of the panel B (cluster 1, n=19 and cluster 0, n=56) for prototypic cytokines GM-CSF, IFNγ, IL-13 and CXCL10. Fisher’s exact test was used to compare the number of cytokine-positive (black) versus negative (grey) patients across clusters. **E.** Barplots representing the percentage and number of patients that will experience a breakthrough COVID-19 infection in the future in each cluster of the Fig. 1B (cluster 0, n=19 and cluster 1, n=56). Fisher’s exact test was used to compare the number of breakthroughs COVID-19 infection-positive (red) versus negative (green) patients across clusters.

Intrigued by the absence of Th1/Tc1 responses against RBD-S1 in 25% of French vaccinees, we conducted a prospective study (‘WELCOME’) using the same immunomonitoring methodology on HCW enrolled at Weill Cornell University (NY, USA). Seventy-five HCW provided blood samples between November 2020 and August 2021 before and after vaccination, with an 21.6±2.12 days-interval between blood sampling and the first vaccine inoculation, and a 44±1.29 days-interval between the vaccination boost and the second blood sampling (**Table S3**, Fig. 1A). Stool sampling allowed to explore the biological significance of intestinal dysbiosis in responses to COVID-19 vaccines. Instead of the 22 hour-whole blood stimulation assay using the VIDAS^®^-30 IFNγ RUO (which requires freshly drawn blood), we determined the capacity of thawed peripheral blood mononuclear cells (PBMCs) to respond to PEPwtRBD by producing 27 distinct Th1/Tc1, Th2, Th17, Th9 and Th10-related cytokines within 72 hours. Ratios between the concentrations of each of the 27 analytes after peptide stimulation (subtracted from the no-stimulation background) were calculated post-versus pre-vaccination for each HCW. We considered responses as positive when the fold ratio between the sample post- and pre-vaccination was superior to 2 (black squares, Fig. 1B). Unsupervised hierarchical clustering of PEPwtRBD-reactivities post-vaccination among the 75 HCW highlighted two distinct clusters (Fig. 1B). In cluster 1, T cell reactivities were mainly driven by Th1 cytokines (GM-CSF, IFNγ, CXCL10, TNFα) accompanied by IL-13 and IL-5 Th2 cytokines (Fig. 1C) that were found in 50–70% individuals (Fig. 1D). Overall, 25% and 40% of HCW also developed a concomitant Th2 response (IL-4, IL-5 respectively) and a small fraction (<20%) secreted inflammatory cytokines or chemokines (G-CSF, CCL4, CCL5) (**Table S3–S4**). Here again, 25% of the individual did not mount any detectable T cell reactivities against PEPwtRBD (cluster 0, Fig. 1B). Of note, co-medications (such as ABX or proton pump inhibitors) could not account for these observed differences (**Table S3**). Among these PEPwtRBD non-responders, 79% also failed to react to PEPmutRBD (**Fig. S2A**), and PEPomRBD (**Fig. S2B**). Similar non-reactivity (79%) was detected against different and larger RBD-S1 peptide residues admixed with membrane and nucleocapsid epitopes (“PEPOrf”)^25^ (**Fig. S2C**). Moreover, two thirds of these individuals were hyporeactive to the mitogen (**Fig. S2D**) and half of them were hyporeactive to peptides common to non-structural proteins (such as helicases and RNA polymerase) of sarbecoviruses^35^ (**Fig. S2E**). Importantly, albeit not significant, the percentage of HCW who was diagnosed with a SARS-CoV-2 breakthrough infection within this timeframe (December 2020-May 2021) was numerically higher in cluster 0 compared with cluster 1 individuals (53% versus 34%, *p*=0.178, Fig. 1E) classified according to PEPwtRBD while it was close-to-random or random for the other peptide pools (PEPOrf: 35% vs 40%, *p*=0.792, PEPmutRBD: 48% vs 35%, *p*=0.319, PEPomRBD: 47% vs 36%, p=0.420 and PEPnsp: 39% vs 39%, *p*=1).

### Associations between gut microbiome composition and PEPwtRBD-specific reactivity in WELCOME.

**Figure 2.**
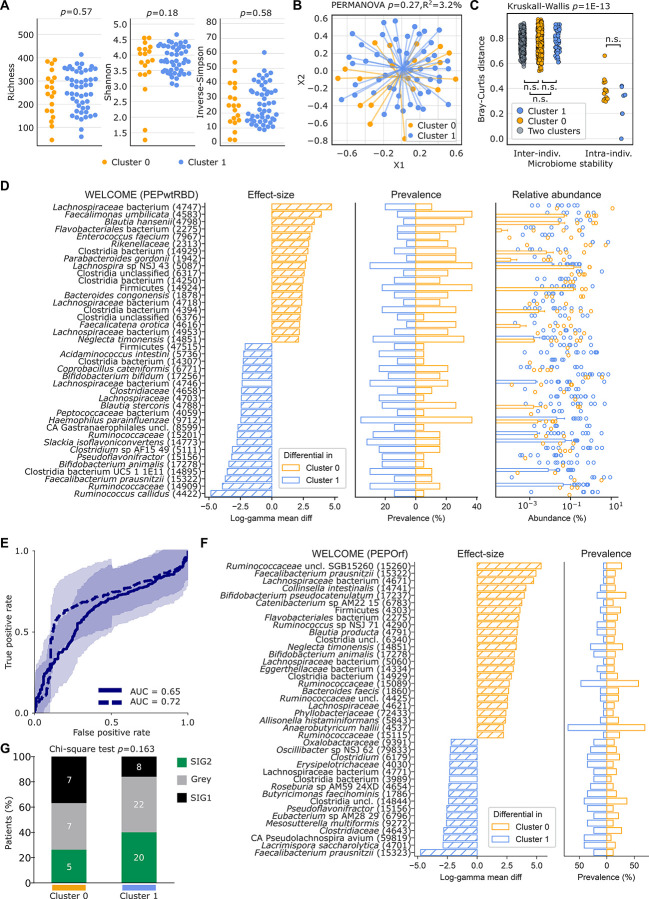
Association between gut taxonomic composition and non-reactivity to COVID-19 Health Care Workers (HCW) vaccines in the WELCOME study. **A.** Microbial species richness, alpha-diversity (log-10-based Shannon entropy), and Inverse-Simpson of the WELCOME cohort microbiome samples (n=69) colored according to the immunoreactivity (orange for cluster 0 or blue for cluster 1). Clusters are defined as PEPwtRBD-specific Th1 reactivity in Fig. 1B. P-values represent Student-t tests. **B.** First two principal components of a multi-dimensional scaling (MDS) based on Bray-Curtis pairwise dissimilarity matrix, colored by immune reaction cluster (orange for cluster 0 or blue for cluster 1 defined in Fig. 1B). P-values are from PERmutational Multivariate ANalysis Of VAriance (PERMANOVA) test. **C.** Intra- vs inter-individual Bray-Curtis pairwise dissimilarities including 69 baseline samples and 22 followed up-samples from the WELCOME cohort. Inter-individual pairs are colored according to the cluster of both samples (orange for cluster 0 or blue for cluster 1 defined in Fig. 1B) or grey for those pairs derived from two different clusters. **D.** Top-40 (absolute values) effect-sizes of a log-link gamma-distributed regression performed on each microbial species-level genome bins (SGB) and filtered for those having *q*<0.2 according to the log-gamma model, colored by sign of the coefficient (orange for cluster 0 or blue for cluster 1, as defined with PEPwtRBD in Fig. 1B). **E.** Ten-times iterated, 10-fold cross-validations ROC curves and AUC of a RF algorithm aiming at distinguishing cluster 1 (reactive) from cluster 0 (non-reactive) as defined with PEPwtRBD in Fig. 1B using all microbiome SGB relative abundances as features, and using the 40 most predictive species as derived by the algorithm internal feature ranking when taking into consideration training folds in order to avoid overfitting. **F.** Top-40 (absolute values) effect-sizes of a log-link gamma-distributed regression performed on each microbial SGB and filtered for those having *q*<0.2 according to the log-gamma model, colored by sign of the coefficient (cluster of association, as defined with PEPOrf in **Fig. S2C**). **G.** Barplots representing the percentage and number of patients belonging to each TOPOSCORE category (SIG2, Grey and SIG1) in each cluster of the Fig. 1B (cluster 0, n=19 and cluster 1, n=56). Fisher’s exact test was used to compare the number of SIG2 (green) versus Grey (grey) versus SIG1 (black) patients in each cluster.

Next, we investigated the relationship between the stool microbiota of HCW in the WELCOME cohort and the presence of post-vaccine Th1/Tc1 immune reactivity to PEPwtRBD. After MG, microbial species composition was estimated using Metagenomic Phylogenetic Analysis 4 (MetaPhlAn 4)^36^ which uses the species-level genome bin (SGB) system^37^ and allows to estimate the relative abundance of both known and yet-to-be-characterized species. The alpha-diversity (calculated using richness, Shannon, and Inverse-Simpson) and beta-diversity (computed on Bray-Curtis pairwise distances) of the microbiota composition in individuals belonging to cluster 0 versus cluster 1 were compared. While cluster 0 versus cluster 1 alpha diversities were not significantly different (Fig. 2A, *p*=0.57, *p*=0.18, *p*=0.58 respectively), beta-diversities tended to differ (Fig. 2B, PERMutational ANalysis Of VAriance (PERMANOVA) *p*=0.27). As an internal control, inter-individual Bray-Curtis distances were superior to those observed intra-individually within 22 HCW before and after vaccination (Kruskall-Wallis *p*<0.001, Dunn-test in all inter- vs. intra- comparisons *q*<0.001) (Fig. 2C).

To determine the relative contribution of each MG species (MGS) relative abundance at baseline to the observed cluster separation, we performed differential relative abundance analyses based on gamma-distributed data regressions with log-link (hereafter termed “log-gamma regression”) on each MGS separately. Species were sorted according to their linear model coefficient after adjustment by age and gender (Fig. 2D). When defined on the basis of responses to PEPwtRBD, cluster 1 of HCW was characterized by the over-representation of several health-associated *Oscillospiraceae* (including *Faecalibacterium prausnitzii* SGB15322, *log-gamma coefficient* (*coeff*.) −3.708 and *q*=0.068, *Ruminococcus callidus* (*log-gamma coeff*. −4.844 and *q*<0.001), *Bifidobacterium* spp. such as *B. animalis, B. bifidum, log-gamma coeff*. −3.411 and *q*=0.049*, log-gamma coeff. −2.347* and *q=*0.082 respectively)*, Lachnospiraceae* (including *Blautia stercoris* (*log-gamma coeff. −2.440* and *q=0.027*)), and the immunogenic *Coprobacillus cateniformis* (belonging to *Erysipelotrichaceae, log-gamma coeff*. −2.304 and *q=*0.019^38^) that were already described in cancer patients responding to immunotherapy^19,23,39–41^ (Fig. 2D, **Table S5a.1**). We then built machine learning algorithms to predict the activation of the immune system using microbiome components only. Ten-times, ten-fold cross-validation receiver operating characteristic (ROC) curves showed an area under the curve (AUC) of 0.65 based on the whole microbiome and 0.72 when taking into consideration the top-40 predictive features (evaluated in training sets-only to avoid overfitting) (Fig. 2E, **Fig. S3A)**. Taking into account the influence of covariates such as gender and age did not ameliorate the performance of the random forest (RF) machine learning in the WELCOME cohort (**Fig. S4**).

Next, we analyzed differences (*q*<0.25) that could contrast the microbial ecosystems of PEPOrf-reactive versus non-reactive HCW (refer to clustering in **Fig. S2C**). The PEPOrf peptide pool encompasses amino acid residues spanning not only the spike (contained in all vaccines), but also the membrane and the nucleocapsid structural proteins both being encountered following SARS-CoV-2 viral events or past history of infections with sarbecoviruses. To better understand the clinical relevance of PEPOrf-specific Th1/Tc1 reactivity against Omicron lineage BA.1, we turned to the COV3APHP cohort that was drawn after the third vaccine boost, just prior to SARS-CoV-2 Omicron outbreak (**Fig. S5A, Table S6**). Here, the clustering of 91 HCW highlighted two groups of subjects (clusters α and β), distinguishable according to the presence or absence of a polyreactive PEPOrf-specific Th1/Tc1 response (IL-5, IL-13, CXCL10, IFNγ, GM-CSF, IL-12p70) (**Fig. S5B-C, Table S6**). HCW exhibiting a mixed Th1/Th2 response were protected against the SARS-CoV-2 Omicron BA.1 viral infection (**Fig. S5D, left panel**; *p*=0.034). At this stage of the pandemics, Th1/Tc1 responses directed against PEPwtRBD were irrelevant for the protection (**Fig. S5D, right panel**). Of note, even after the SARS-CoV-2 Omicron BA.1 wave which infected 40/79 HCW (51%), non-reactivity to PEPomRBD was observed in 29% of individuals (**Fig. S5E, left panel**) that failed to coincide with the history of Omicron BA.1 infection (**Fig. S5E, right panel**).

As shown for the PEPwtRBD reactivity, the strongest association between PEPOrf-induced T-cell responses and microbial species were observed for *Lachnospiraceae* family members (such as *Lacrimispora saccharolytica* (*log-gamma coeff*. −3.411 and *q*=0.100, *Roseburia* spp. AM59 24XD (*log-gamma coeff*. −2.375 and *q*=0.125), *Faecalibacterium prausnitzii* (SGB15323, *log-gamma coeff*. −4.753 and *q*<0.001),) and *Erysipelotrichaceae* spp. Moreover, immunosuppressive bacteria (such as *Flavobacteriales* and *Eggerthellaceae*) were once again found to be associated with the absence of PEPOrf responses (cluster 0) (Fig. 2F, **Table S5a.2**). Admittedly, cluster 0 individuals also presented with health-related species such as *Faecalibacterium prausnitzii* (SGB15322), *Lachnospiraceae bacterium* or *Ruminococcaceae* unclass. Therefore, using a second independent method taking into account bacteria ecological topology-based tool TOPOSCORE we confirmed that the frequency of SIG1 individuals tended to be reduced in cluster 1 compared to cluster 0 vaccinees (Fig. 2G, *p*=0.16).

Altogether, we conclude that a gut microbiota repertoire deviated from the healthy composition, could at least in part, explain the poor immunogenicity of vaccines observed in 25% of individuals.

### Stool microbial taxonomic composition and vaccine antigen-specific delayed-type hypersensitivity (DTH) responses in the MIND-DC trial.

**Figure 3.**
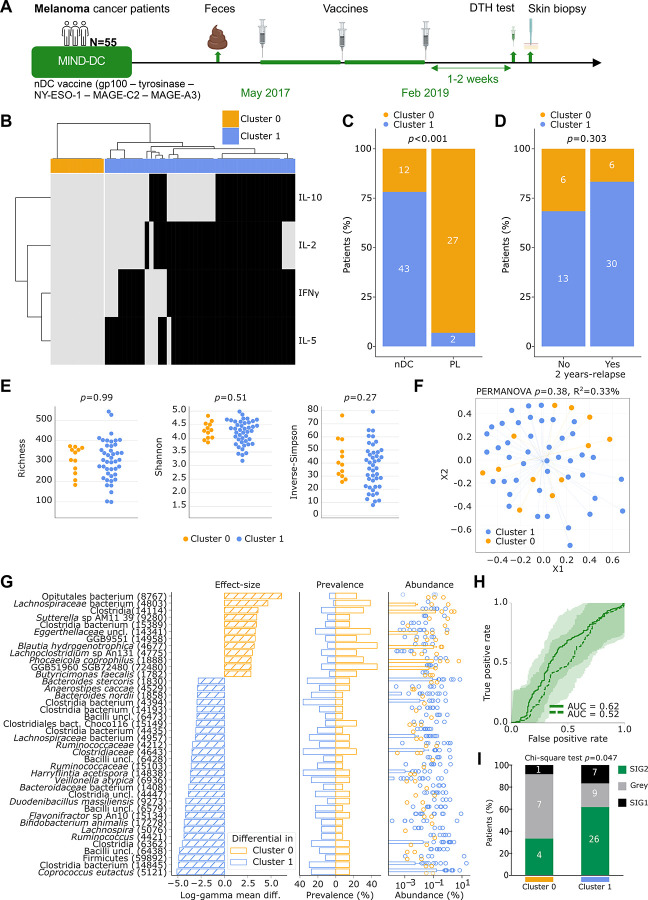
Association between gut taxonomic composition and immunoreactivity to adjuvant treatment with dendritic cell (DC) therapy in patients with melanoma. **A.** Graphical representation of the MIND-DC cohort protocol with fecal and skin biopsy collection and timelines between vaccination and sampling (refer to **Table S3**). **B.** Bicolor map of cytokine secretion after delayed-type hypersensitivity (DTH) skin test (positive in black, negative in grey). Patients (n=55) were ordered in columns and cytokines were ordered in rows by unsupervised hierarchical clustering using ward.D2 clustering method and binary distance calculation method. Heatmap was cut in 2 clusters of patients (orange: non-reactive (cluster 0), blue: polyreactive (cluster 1)). **C.** Barplots representing the percentage and number of patients that are IL-10- and/or IL-2- and/or IFNγ- and/or IL-5-reactive/areactive in DTH responses in both autologous natural DC-vaccinated and placebo (PL) groups (orange: cluster 0, blue: cluster 1 as defined in Fig. 3B). Fisher’s exact test was used to compare the number of cytokinepositive patients across groups. **D.** Barplots representing the percentages and number of patients from cluster 0 (orange) and 1 (blue) as defined in Fig. 3B in patients with or without tumor relapse at 2 years. Fisher’s exact test was used to compare percentages in both clusters. **E.** Microbial species richness, alpha-diversity (log-10-based Shannon entropy), and Inverse-Simpson of the MIND-DC cohort microbiome samples colored by immune reaction cluster (orange for cluster 0 or blue for cluster 1). Clusters are defined in as defined in Fig. 3B (cluster 0 versus 1). P-values are from Student-t tests. **F.** First two principal components of a multi-dimensional scaling (MDS) based on Bray-Curtis pairwise dissimilarity matrix, colored by immune reaction (cluster 0 vs 1 defined in Fig. 3B). P-values are from PERmutational Multivariate ANalysis Of VAriance (PERMANOVA) test. **G.** Top-40 (absolute values) effect-sizes of a log-link gamma-distributed regression performed on each microbial species-level genome bins (SGB) and filtered for those having *q*<0.2 according to the log-gamma model, colored by sign of the coefficient (orange for cluster 0 or blue for cluster 1, as defined in Fig. 3B). **H.** Ten-times iterated, 10-fold cross-validations ROC curves and AUC of a RF algorithm aiming at distinguishing cluster 1 (reactive) from cluster 0 (non-reactive) from panel B using all microbiome SGB relative abundances as features, and using the 40 most predictive species as derived by the algorithm internal feature ranking when taking into consideration training folds in order to avoid overfitting. **I.** Barplots representing the percentage and number of patients belonging to each TOPOSCORE category (SIG2, Grey and SIG1) in each cluster of the Fig. 3B (cluster 0, n=12 and cluster 1, n=43). Fisher’s exact test was used to compare the number of SIG2 (green) versus Grey (grey) versus SIG1 (black) patients in each cluster.

To further investigate links between the vaccine-induced immune tonus and a favorable microbiome community structure, we took advantage of a cohort of patients with stage IIIB/IIIC cutaneous melanoma vaccinated with peptide-pulsed autologous natural DC (nDC)^22^. The MIND-DC phase III clinical trial (NCT02993315) randomized 148 patients with melanoma in a 2:1 ratio to adjuvant treatment with nDC (pulsed with gp100, tyrosinase, NY-ESO-1, MAGE-C2 and MAGE-3 melanoma derived peptides, which are tumor associated antigens, TAA) versus placebo^22^ (Fig. 3A, **Table S3**). MG-based sequencing of stool samples revealed that the intestinal microbiota composition was weakly associated with 2-year recurrence in the whole cohort, regardless of the treatment arm^23^. Patients receiving nDC exhibited a delayed type hypersensitivity (DTH) response to DC loaded with the TAA injected into the skin, while patients of the placebo arm did not. We examined the pattern of cytokine release by skin-infiltrating lymphocytes (from the site of DTH response) restimulated *ex vivo* with TAA in 57 nDC vaccinated patients (active arm) for whom stool MG data were available^23^ and 29 placebo-treated patients (placebo arm). Both stool and skin samples were collected in 55 patients before 3 biweekly nDC injections to allow correlations between gut taxa at baseline and DTH T cell responses post-nDC vaccination (Fig. 3A, **Table S3**). Four cytokines were monitored, allowing to segregate these patients into 2 clusters using unsupervised hierarchical clustering (Fig. 3B). As shown for HCW, 22% of patients within the nDC arm were non-reactive, defined as failed DTH response or absent cytokine release by TAA-treated lymphocytes (Fig. 3B–C). Of note, 93% of the patients enrolled in the placebo arm were non-reactive (Fig. 3C). However, nDC-based Th1/Tc1 immunization was not clinically relevant against relapse in this trial (Fig. 3D, *p*=0.303)^22^.

Despite that, we compared the intestinal microbiota of immune-reactive (cluster 1) versus non-reactive (cluster 0) patients. The alpha diversity (calculated using gene richness (*p*=0.99), Shannon (*p*=0.51), and Inverse-Simpson (*p*=0.27)) and the beta diversity (PERMANOVA, *p*=0.38) of the microbiota composition in melanoma patients belonging to cluster 0 versus cluster 1 were not different (Fig. 3E–F, **Table S5b**). In the next step, we assessed the statistical association of immune responses to MGS relative abundance using log-gamma regression models adjusted by patients’ gender and age. Several beneficial and health-associated MGS were over-represented in immunoreactive individuals, including multiple *Lachnospiraceae* and *Oscillospiraceae* family members (*Coprococcus eutactus (log-gamma coeff*. −5.108 and *q*<0.001)*, Ruminococcus* SGB4421 (*log-gamma coeff*. −4.341 and *q*=0.002), *Lachnospira* SGB5076 (*log-gamma coeff*. −4.341 and *q*=0.002), *Ruminococcaceae* SGB15103 (*log-gamma coeff*. −3.6 and *q*=0.077), *Ruminococcaceae* SGB4212 (*log-gamma coeff*. −3.424 and *q*=0.009), and *Bifidobacterium animalis (log-gamma coeff*. −4.319 and *q*=0.121), (Figure 3G, **Table S5b**). Ninety-one species in total were found to be associated with a high immune tonus (*q*<0.2). These species were found to be increased at the relative expense of 49 bacteria associated to the absence of immune responses (such as *Eggerthellaceae* and *Proteobacteria*^21^). Ten-times, ten-fold cross-validation ROC curves showed an AUC of 0.62 for MIND-DC based on the whole microbiome (Fig. 3H). Including sex and age as features did not improve the performance of the RF machine learning (**Fig. S4**). Using a second independent method taking into account bacteria ecological topology “the TOPOSCORE”, we confirmed that the frequency of SIG2 individuals tended to be increased in cluster 1 compared to cluster 0 vaccinees (Fig. 3I, *p*=0.047).

Altogether, these results indicate that intestinal dysbiosis correlates with poor responses to a DC-based vaccine in melanoma patients.

### Stool microbial taxonomic composition and telomerase antigen-specific Th1 responses in the UCPVax trial.

**Figure 4.**
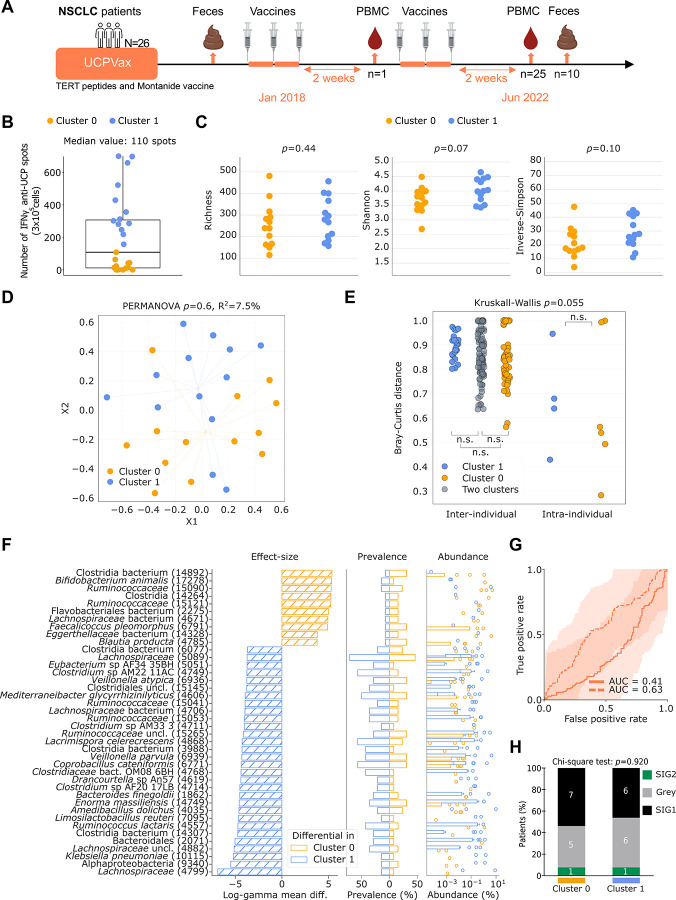
Association between gut taxonomic composition and immunoreactivity to UCPVax in non-small cell lung cancer (NSCLC) patients. **A.** Graphical representation of the UCPVax cohort protocol with fecal and blood collection and timelines between vaccination and sampling (refer to **Table S3**). TERT, telomerase reverse transcriptase. **B.** Boxplot representing the number of IFNγ anti-UCP spots (for 3.10^5^ cells). Each point represents a patient. The middle line of the box represents the median and equals to 110 spots. The lower and upper hinges correspond to the first and third quartiles (the 25th and 75th percentiles). The upper whisker extends from the hinge to the largest value no further than 1.5 × IQR from the hinge (where IQR is the interquartile range, or distance between the first and third quartiles). The lower whisker extends from the hinge to the smallest value at most 1.5 × IQR of the hinge. Data beyond the end of the whiskers are called “outlying” points and are plotted individually. Colors are set depending on IFNγ-reactivity: orange stands for non-reactive patients (“cluster 0” if less than 110 spots) and blue stands for immunoreactive patients (“cluster 1” if more than 110 spots). Refer to **Table S7**. **C.** Microbial species richness, alpha-diversity (log-10-based Shannon entropy), and Inverse-Simpson of the UCPVax cohort microbiome samples colored by immune reaction cluster (orange for cluster 0 or blue for cluster 1). Clusters are defined in Fig. 4B. P-values are from Student-t tests. **D.** First two principal components of a multi-dimensional scaling (MDS) based on Bray-Curtis pairwise dissimilarity matrix, colored by immune reaction clusters defined in Fig. 4B (orange for cluster 0 or blue for cluster 1). P-values are from PERmutational Multivariate ANalysis Of VAriance (PERMANOVA) test. **E.** Intra- vs inter-individual Bray-Curtis pairwise dissimilarities considering 26 baseline samples as well as 10 followed up samples from the UCPVax cohort. Inter-individual pairs are colored according to the cluster of both samples (orange for cluster 0 or blue for cluster 1 defined in Fig. 4B) or grey for pairs derived from two different clusters. **F.** Top-40 (absolute values) effect-sizes of a log-link gamma-distributed regression performed on each microbial species-level genome bins (SGB) and filtered for those having *q*<0.2 according to the log-gamma model, colored by sign of the coefficient (“cluster” of association, as defined in panel B). **G** Ten-times iterated, 10-fold cross-validations ROC curves and AUC of a random forest (RF) algorithm aiming at distinguishing “cluster 1” (reactive) from “cluster 0” (non-reactive) using all microbiome SGB relative abundances as features, and using the 40 most predictive species as derived by the algorithm internal feature ranking when taking into consideration training folds in order to avoid overfitting. **H.** Barplots representing the percentage and number of patients belonging to each TOPOSCORE category (SIG2, Grey and SIG1) in each cluster of the Fig. 4B (cluster 0, n=12 and cluster 1, n=43). Fisher’s exact test was used to compare the number of SIG2 (green) versus Grey (grey) versus SIG1 (black) patients in each cluster.

To extend findings obtained in HCW and melanoma patients, we next analyzed samples from the UCPVax Phase Ib/IIa clinical trial. This trial enrolled patients with stage IV non-small cell lung cancer (NSCLC) that were treated with universal cancer peptide-based vaccine (UCPVax) that is based on human telomerase reverse transcriptase (hTERT) derived-MHC class II peptides capable of eliciting CD4^+^ Th1 responses^24^. In this trial, the induction of anti-hTERT immunity significantly correlated with survival in NSCLC patients refractory to chemotherapy or immunotherapy with programmed cell death protein 1 (PD-1) blockade^24^. Baseline and post-vaccine stools with paired PBMC (harvested after 6 priming vaccines) were available in 26 and 10 NSCLC patients respectively (Fig. 4A, **Table S3**). We previously reported that all immune responders after the first three injections (n=51) maintained or improved anti-hTERT CD4^+^ T-cell responses after six immunizations (n=39)^24^. In the current study, we analyzed metagenomic differences between two subgroups of NSCLC patients split according to the median value of hTERT-specific IFNγ secreting T lymphocytes (110 spots per 3×10^5^ PBMC detectable by mean of an enzyme-linked immunosorbent spot (by ex vivo ELISPOT assay) (**Table S5c, Table S7,** Fig. 4B). This median was not significantly different from the one obtained in the whole cohort previously described^24^ and patients exhibiting >110 hTERT-specific spots were considered as “immunoreactive” (“cluster 1”, n=13), while patients with <110 hTERT-specific spots were considered as non-reactive (“cluster 0”, n=13). This latter group of patients exhibited a higher proportion of adenocarcinoma than the immunoreactive group (**Table S3**).

As shown for the two previous cohorts, there was no significant difference in alpha-(calculated using richness *p=*0.44, Shannon *p=*0.07 and Inverse-Simpson *p=*0.10, Fig. 4C) and beta-diversities between these 2 groups (PERMANOVA, *p*=0.6 Fig. 4D). Of note, there was a relative stability of the fecal phenotype with no significant intra-individual evolution of the beta-diversity between baseline and post-vaccine stool metagenomics composition in 10 NSCLC patients sampled twice compared with the inter-individual variations, in both “clusters” (Kruskall-Wallis *p*=0.055, Fig. 4E). To determine if distinct MGS could contribute to the separation of both “clusters” defined using the ELISPOT immunoreactivity after 6 vaccine shots, log-gamma regression models were run on each species to determine differential relative abundance across “clusters”, while adjusting for gender and age (Fig. 4F). Out of 626 species, 162 were associated with the immunoreactive “cluster 1” and 75 were associated with the nonreactive “cluster 0” (*q*<0.2). Distinct MGS that were associated with immune non-reactivity post-hTERT vaccine were already described in the former cohorts (such as *Eggerthellaceae* (*log-gamma coeff*. 3.800 and *q*=0.004) and *Flavobacteriales* (*log-gamma coeff*. 4.991 and *q*<0.001)) and were associated with poor prognosis in NSCLC patients taking ABX or resistant to PD-1/PD-L1 blockade^42^. The majority of the significant associations were found within the immunoreactive “cluster 1”, including several *Lachnospiraceae* (*Lacrimispora celerecrescens*, *log-gamma coeff*. −4.267 and *q*<0.001, *Lachnospiraceae* SGB4799, *log-gamma coeff*. −6.883 and *q*<0.001) and *Oscillospiraceae* family members (such as *Ruminococcus lactaris, log-gamma coeff*. −4.724 and *q*<0.001) and the immunogenic *Coprobacillus cateniformis, log-gamma coeff*. −4.365 and *q*<0.001^38^ (Fig. 4F). Admittedly, cluster 1 individuals were also harboring pathobionts (*Klebsiella pneumoniae*, *Alphaproteobacteria*) that may restrain vaccine immunogenicity. Machine learning partially confirmed the trend observed at the single-species level, as the top-40 discriminant features (extracted from the training sets-only to avoid overfitting) were predictive of immune reactivity in the UCPVax cohort at an AUC of 0.63 (Fig. 4G; **Fig. S3C**). Using a second independent method taking into account bacteria ecological topology “the TOPOSCORE”, we confirmed that this population of patients with lung cancer treated from second line was very dysbiotic (with no SIG2 subset) explaining the poor prediction of the model (Fig. 4H, *p*=0.92).

### Meta-analysis of the association between intestinal taxonomic composition and failure of viral or cancer vaccines.

**Figure 5.**
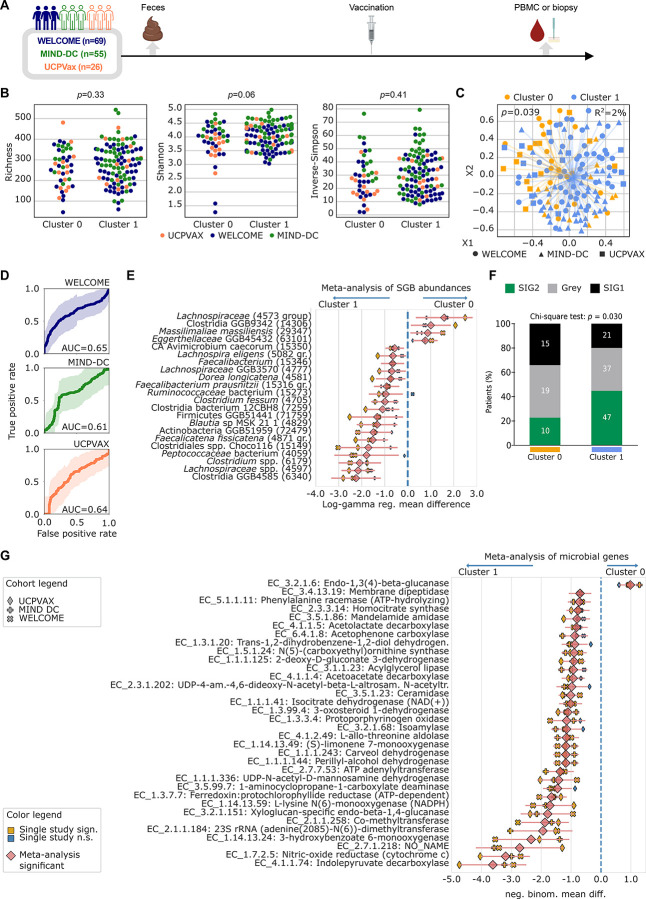
Meta-analysis of the association between gut taxonomic composition and immunoreactivity to vaccines. **A.** Graphical representation of the meta-analysis encompassing three cohorts (WELCOME, MIND-DC and UCPVax, n=150). **B.** Microbial species richness, alpha-diversity (log-10-based Shannon entropy), and Inverse-Simpson of the 3 cohorts’ microbiome samples colored by cohort (blue for WELCOME, green for MIND-DC and orange for UCPVax). P-values are from Student-t tests. **C.** First two principal components of a multi-dimensional scaling (MDS) based on Bray-Curtis pairwise dissimilarities, colored by immune reaction clusters (orange: non-reactive (cluster 0), light blue: reactive (cluster 1)) of the pooled three cohorts; symbols indicate the three cohorts. P-values are from PERmutational Multivariate ANalysis Of VAriance (PERMANOVA) test. **D.** Ten-times iterated, 10-fold cross-validations ROC curves and AUC of a random forest (RF) algorithm aiming at distinguishing cluster 1 (reactive) from cluster 0 (non-reactive) of the pooled three cohorts using all microbiome species-level genome bins (SGBs) relative abundances as features in each cohort independently while microbiome SGBs relative abundances of the other two cohorts are added to each training folds during the training phase. **E.** Condensed Forest plot on the twenty-two microbial SGBs’ showing the highest coefficient (absolute value) from a meta-analysis of log-gamma regression models and found at *q*<0.2 according to the same meta-analysis and in all three cohorts (sign of the coefficient corresponds to the cluster of association of the pooled three cohorts). **F.** Barplots representing the percentage and number of patients belonging to each TOPOSCORE category (SIG2, Grey and SIG1) in each cluster (cluster 0 and cluster 1 as defined in Fig. 1B, 3B, 4B) in the pooled cohorts. Fisher’s exact test was used to compare the number of SIG2 (green) versus Grey (grey) versus SIG1 (black) patients in each cluster. **G.** Condensed Forest plot on the forty microbial enzymes showing the highest coefficient (absolute value) from a meta-analysis of negative binomial distributed regression models and found at *q*<0.2 according to the same meta-analysis and in all three cohorts (sign of the coefficient corresponds to the cluster of association of the pooled three cohorts).

To discover commonalities in the microbiota characteristics associated with poor Th1 immune response, we pooled the three cohorts and performed joint analyses (Fig. 5A). We pooled all cluster 0 and cluster 1 patients of the three cohorts and analyzed their alpha- and beta-diversities. The alpha-diversity was not superior in immunoreactive (cluster 1) versus non-reactive (cluster 0) individuals (microbial richness (*p*=0.33), Shannon (*p*=0.06), and Inverse-Simpson (*p*=0.41, Fig. 5B), while beta-diversity was significantly different between the two clusters (PERMANOVA *p*=0.039, Fig. 5C). We then used machine learning models, which were trained and tested in cross-validation on each cohort separately, aiming to predict clusters of immune activation based on microbiome composition. When attempting predictions on one cohort, the other two cohorts were iteratively added in the training. Using this approach, we obtained similar or better performances with AUC values reaching 0.65, 0.61 and 0.64 for WELCOME, MIND-DC and UCPVax cohorts, respectively, suggesting that the three cohortbased predictions obey to similar microbiome community patterns (Fig. 5D).

To explore which microbiome members are mostly representative of the “reactive” versus “non-reactive” immune phenotype post-vaccine, we pooled the log-gamma models employed and performed a meta-analysis of regression models to identify MGS relative abundances that are significantly increased in either cluster 1 or 0. Four species were associated with the non-reactive cluster: the *Lachnospiraceae* SGB4573, the Clostridia SGB14306, *Massilimaliae massiliensis* (already described in patients with melanoma and/or NSCLC exhibiting progressive disease despite PD-1 and/or cytotoxic T-lymphocyte associated protein 4 (CTLA-4) blockade^21^), and the *Eggerthellaceae* SGB63101 (*log-gamma average (avg.) coeffs*. 1.5846, 1.002, 0.868, and 0.765, respectively and meta-analysis *q*<0.25). Eighteen species were associated with immunoreactivity observed in cluster 1 of all three cohorts. Among those, distinct *Lachnospiraceae* family members were already observed in responders to cancer immunotherapy^21^, such as *Faecalicatena fissicatena, Dorea longicatena, Faecalibacterium prausnitzii* (SGB15316), and *Lachnospira eligens* (*log-gamma avg. coeff.* −1.519 and meta-analysis *q*<0.001, *log-gamma avg. coeff*. −0.742 and meta-analysis *q*=0.245, *log-gamma avg. coeff*. −0.915 and meta-analysis *q*=0.025 and *log-gamma avg. coeff*. −0.647 and meta-analysis *q*=0.247, respectively). Other species did result in a significant association with the three cohorts pooled together while they were not detected in single-cohort analyses (i.e. *Clostridium fessum*, *log-gamma avg. coeff*. −0.992, *q*=0.026), suggesting a more general mechanism of microbiome-mediated immune activation mechanisms (Fig. 5E). In addition, the strongest associations found were with uncharacterized SGBs, such as the *Clostridia* SGB6340 in the reactive cluster (*log-gamma avg. coeff*. −2.246, *q*<0.001), and the *Lachnospiraceae* SGB4573 group in the non-reactive cluster (*log-gamma avg. coeff*. 1.585, *q*=0.177) (Fig. 5E), calling for investigation of this thus far uncultured species. Using a second independent method taking into account bacteria ecological topology “the TOPOSCORE”, we confirmed that the frequency of SIG2 individuals in the metaanalysis was significantly increased in cluster 1 compared to cluster 0 vaccinees (Fig. 5F, *p*=0.027).

It is interesting to note that the RF machine learning per cohort adding the two other cohorts predicted the top-40 feature variable importance, most of these features in each cohort being confirmed in this meta-analysis (**Fig. S3**). All three cohorts’ cluster 0 and 1 were primarily separated by *Clostridium fessum, Faecalicatena fissicatena, Dorea longicatena, Faecalibacterium prausnitzii* SGB15316 and other species identified in multi-cohorts and in single-cohort analyses. Of note, univariate analyses associating prevalence of metagenomic species with overall survival within a large cohort of 498 NSCLC patients already reported^21^ and in a MIND-DC melanoma cohort^22,23^ highlighted the clinical relevance of *Faecalibacterium prausnitzii* SGB15316 but not that of *F. prausnitzii* SGB15322 nor SGB15323 (**Fig. S6**).

To explore putative microbial functions distinguishing clusters 0 and 1, we applied the HUMAnN 3.8 pipeline to the metagenomic data. This pipeline first annotated a total of 3,144,604 UniRef90 gene families, further grouped into 2,540 Enzyme Commission (EC) number entries. By performing a meta-analysis of negative binomial family regression models (see Methods), we found a total of 61 genes that were associated with cluster 1 of immune activation (meta-analysis *q*<0.1), and one gene associated with cluster 0 (non-reactive), when meta-analyzing the three cohorts together (n=156) (Figure 5G). When selecting only those found at *q*<0.01, cluster 1-associated metabolic functions (33 in total) encompassed indolepyruvate decarboxylase, a key enzyme in indole-3-acetic acid biosynthesis^43^ (EC 4.1.1.74, *negative bin. coeff*. −3.6, meta-analysis *q*<0.001) involved in a beneficial resetting of the tumor microenvironment and progression-free survival in cancer patients^44^. Microbial gene-families encoding nitric oxide reductase were also relatively over-represented in cluster 1 (EC 1.7.2.5, *coeff*. −3.19, *q*<0.001), as reported in gastric cancer patients after stomach partial surgery where a remodeling of the gastric microbiota was observed^45^. Isocitrate dehydrogenase, also dominant in cluster 1 (EC 1.1.1.41, *coeff*. −1.01, *q*<0.001), catalyzes the decarboxylation of isocitrate into α-ketoglutarate which synergizes with PD-1 blockade in tumor models^46^. Another trait of cluster 1 is the presence of acetoacetate decarboxylase (EC 4.1.1.4, *coeff*. −0.93, *q*<0.001) which is an immunostimulation-mediating ketogenesis enzyme^47^. Moreover, oxidoreductases (dehydrogenases, monooxygenases, lipase for NADPH cofactor regeneration) represented 20 out 61 of the genes associated with cluster 1 at *q*<0.1 (33% compared to 20% of the total genes analyzed [363 out of 1,791], Chi-squared statistic 4.9, Chi-squared test *p*=0.027) and constitute one of the most important free radical scavenger systems involved in many physiological processes.

Altogether, this meta-analysis reveals compositional differences between cluster 0 and cluster 1 centered on key species and metabolic pathways associated with immunostimulation.

### Gut microbiota and humoral immune responses to COVID19 vaccine

**Figure 6.**
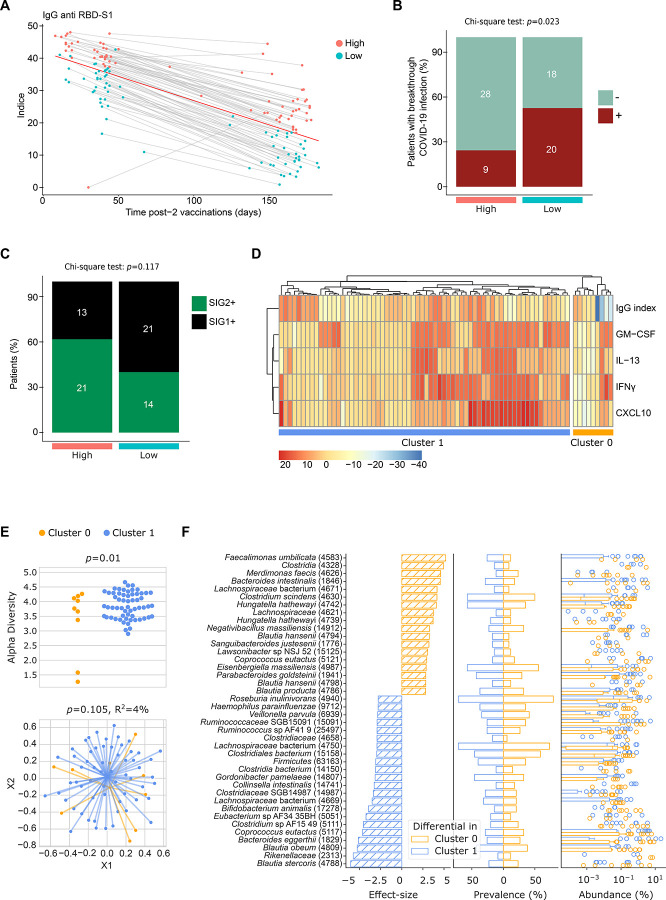
Gut microbiota fingerprint associated with defects in both humoral and cellular immune responses to COVID19 vaccine in the WELCOME cohort. **A.** RBD-S1 specific IgG titers were determined after the prime-boost COVID19 vaccine in 75 HCW from WELCOME at two timepoints between the prime boost and 200 days after (refer to Fig. 1A). The cohort was divided according to the median of the decay of antibody titers into two groups “low” (if lower to the median) versus “high” (above the median). **B.** Barplots representing the percentage and number of patients that will experience a breakthrough COVID-19 infection in the future in humoral immune responses’ groups of the Fig. 6A. P-value represent Chi2 test. **C.** Barplots representing the percentage and number of patients belonging to each TOPOSCORE category (SIG2, Grey and SIG1) in each cluster (high and low as defined in Fig. 6A). Chi2 test was used to compare the number of SIG2+ (green) versus SIG1+ (black) patients in each humoral immune responses’ groups**. D.** Unsupervised hierarchical clustering of the all health care workers (HCW) according to the PEPRBDwt-specific Th1 cytokine release and RBD-S1-specific IgG titers, revealing two clusters, cluster 0 and cluster 1 for “low” and “high” combined reactivities, respectively. **E**. Upper panel represents microbial species alpha-diversity (log-10-based Shannon entropy) of the WELCOME cohort microbiome samples colored by cluster (orange for cluster 0 and blue for cluster 1). P-values are from Student-t tests. Lower panel represents first two principal components of a multi-dimensional scaling (MDS) based on Bray-Curtis pairwise dissimilarities, colored by immune reaction clusters (orange: non-reactive (cluster 0), light blue: reactive (cluster 1)) of the WELCOME cohort. P-values are from PERmutational Multivariate ANalysis Of VAriance (PERMANOVA) test. **F.** Top-40 (absolute values) effect-sizes of a log-link gamma-distributed regression performed on each microbial species-level genome bins (SGBs) and filtered for those having q<0.2 according to the log-gamma model, colored by sign of the coefficient (cluster of association, as defined in Fig. 1D).

We next analyzed the impact of the gut taxonomic composition on the RBD-S1-specific antibody titers. Two longitudinal serum sampling in all HCW from the WELCOME cohort allowed careful monitoring of seroconversion against the RBD-S1 protein, pre- and post - prime/boost and third COVID 19 vaccine, as shown Fig. 1A as well as collection of intercurrent breakthrough infections. Using the median of the decay of antibody titers, we could segregate the WELCOME cohort in two subgroups (“high” versus “low” antibody levels overtime) (Fig. 6A) that correlated with subsequent viral infections (Fig. 6B, *p*=0.023). Of note, there was no other difference between these two seroconverted groups of WELCOME except age, older HCW exhibiting lower antibody titers (**Table S3b**). Albeit not statistically significant, SIG2+ patients were numerically higher among HCW reaching high RBD-S1-specific Ab titers (Fig. 6C, *p*=0.117). There was no correlation between humoral and cellular immune responses (as described in Fig. 1B) in the WELCOME cohort (*p*=0.111). Therefore, we combined both parameters and performed an unsupervised hierarchical clustering of WELCOME individuals tested in both assays and found a small subgroup of 9 out 69 who did not perform well in both assays (Fig. 6D). The analysis of the alpha and beta diversity of the intestinal ecosystem in these 9 HCW compared with the rest of the cohort showed a reduced richness (Alpha diversity *p*=0.01, Fig. 6E) and a slight difference in the stool composition (*p*=0.105, Fig. 6F) dominated by ABX-associated pathobionts (such as *Hungatella hathewayi* SGB4742 and SGB4739)^48^ and high fat diet *(Eisenbergiella massiliensis*)^47^ (Fig. 6G). Altogether, combining both humoral and cellular immune responses, we confirmed that about 13% of HCW, characterized by a relative gut dysbiosis, could not fully benefit from vaccine-induced immunization.

To re-assess the potential impact of the gut microbiota composition on humoral immune responses, we analyzed another cohort of healthy volunteers from Texas, US, the “EnDVR (Environmental Determinants of Vaccine Response) Study” (NCT05239403, **Table S8)**. The study was initiated in 2021 to investigate whether fecal microbiome composition and dietary habits correlate with vaccine response to influenza virus. After the approval of the first COVID-19 vaccines in December 2020, the study was expanded to include antibody measurements directed against SARS-CoV-2 spike protein using a reference ELISA method^49^. Shot gun metagenomic sequencing at baseline and antibody titers directed against SARS-CoV-2 spike protein pre- and post-booster (3rd shot) vaccination information was available in 49 HV. The correlation between the fecal taxonomic profiling and the effect of the booster on the rise in Ab titers could only be performed in 35 individuals (**Fig. S7A**), either because no booster was received or because the serum sampling was preceding the booster. 21 HV (60%, **Table S8**) responded to the booster (cluster 1) while 14 failed to respond (cluster 0). The stool richness tended to be superior in cluster 1 (vs cluster 0) HV (p=0.08, **Fig. S7B left panel**). Although beta-diversity was not significantly different between the 2 groups (**Fig. S7B, right panel**), there was a relative enrichment of Lachnospiraceae family members (*Blautia spp., Chlostridiaceae, Christensenella hongkongensis, Simiaoa spp*.) and *Ruminococcus torques* in cluster 1 while individuals in cluster 0 had a relative enrichment of oral “immunosuppressive or proinflammatory” taxa (*Veillonella dispar, Veillonella atypica, Rikenellaceae bacterium*) (**Fig. S8C**). Toposcore analysis revealed a trend towards a positive correlation between the S score and the relative increase in Ab titers in cluster 1 but not cluster 0 (**Fig. S8D**), as well as a trend towards a higher fraction of SIG2+ in cluster 1 (47% SIG2+ in cluster 1 vs 28% SIG2+ in cluster 0, p=0.4) (**Fig. S8E**). In this small study of healthy volunteers receiving SARS-CoV-2 booster vaccines, 40% showed no significant increase in antibody levels. These nonresponders also had lower microbiome diversity but more inflammatory markers in their stool samples compared to vaccine booster responders, suggesting a link between gut health and vaccine response.

### Gut microbiota and live attenuated yellow fever 17D vaccine strain (YF17D) vaccine

**Figure 7.**
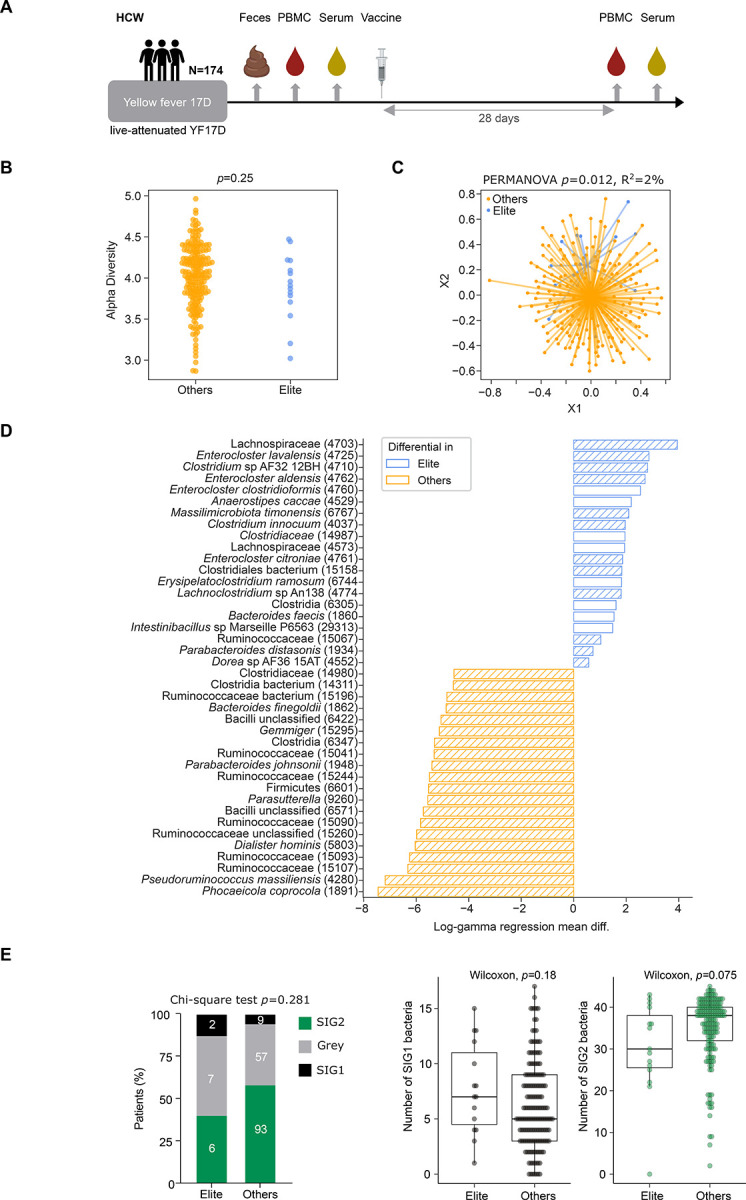
Elite responders to YF17D vaccine harbor an immunosuppressive intestinal microbiota. **A.** Graphical representation of the study comprising young healthy volunteers vaccinated with the live replicative YF17D vaccine (n=174). Serum, PBMC and stools were drawn before and 28 days after the immunization. Elite vaccine responders (n=15) were previously defined^51^, performing above average in all three categories neutralizing antibody titer and YFV17D specific CD4 and CD8 T cell responses. **B.** Microbial species alpha-diversity (log-10-based Shannon entropy) of the YF17D cohort microbiome samples colored by cluster (blue for elite and orange for others). P-values are from Student-t tests. **C.** First two principal components of a multi-dimensional scaling (MDS) based on Bray-Curtis pairwise dissimilarities, colored by immune reaction groups (blue: elite; orange: others) of the YF17D cohort. P-values are from PERmutational Multivariate ANalysis Of VAriance (PERMANOVA) test. **D.** Top-40 (absolute values) effect-sizes of a log-link gamma-distributed regression performed on each microbial species-level genome bins (SGB) and filtered for those having *q*<0.2 according to the loggamma model, colored by sign of the coefficient (elite (n=15, blue) and non-elite (n=159, orange) vaccine responders as previously defined^51^. **E.** Barplots representing the percentage and number of patients belonging to each TOPOSCORE category (SIG2, Grey and SIG1, left panel) or to the absolute numbers of prevalent SIG1 and SIG2 bacteria (right panel) in elite (n=15) and non-elite (n=159) vaccine responders as previously defined^51^. Fisher’s exact test was used to compare the number of SIG2 (green) versus Grey (grey) versus SIG1 (black) patients in each cluster.

In contrast to the vaccines studied above, the excellent performance of the YF17D live attenuated vaccine to elicit long lasting protective immunity may explain the scarcity of research studies evaluating intrinsic or extrinsic causal factors modulating its efficacy^50^. A recent report on 250 healthy young adults analyzed the baseline immune parameters associated with cellular (YF17D-specific CD4^+^ and CD8^+^ T cells) and humoral (YF17D-specific neutralizing antibody titers) immune responses^51^. In this report, 250 healthy young individuals (81 males, 169 females, age 19–47 years, median 24y) were vaccinated with the Stamaril^®^ YF17D vaccine. For 174 of them, stool samples at baseline (before YF17D vaccination) could be paired with PBMC and serum samples from baseline and day 28 post-vaccination. YF17D-specific IgM and IgG, and neutralising antibody titers, as well as the functionality and the frequency of YF17D-specific CD4^+^ and CD8^+^ T cell responses were analysed and have been recently reported^51^. The functionality score of T cells was calculated using a Combinatorial Polyfunctionality Analysis of Single Cells (COMPASS) analysis^52^. The YF17D vaccine is highly immunogenic^53^, and all study participants developed neutralizing antibodies and antigen-specific T cells by day 28 post-immunization^51^. To divide vaccinees into good, average and poor responders, Santos et al. performed hybrid hierarchical k-means clustering of the three main vaccination endpoints, the frequency and functionality of YF17D-specific CD4^+^ and CD8^+^ T cells, and the neutralizing antibody titer^51^. Individuals present in the good responder group for each of the three endpoint categories were classified in a composite score as “elite” vaccine responders^51^. Indeed, Santos-Peral et al. describe elevated numbers of cytokine-expressing T helper cells in circulation at baseline to be associated with stronger vaccine responses. However with the exception of smoking we did not detect a direct association between the tested independent factors modulating immune parameters at baseline including sex, Cytomegalovirus (CMV) status, and exposure to other previous infections, and the response to the YF17D vaccine on day 28 in our previous report^51^. Therefore, in this manuscript, we analyzed potential links between the stool taxonomic composition at baseline and the response status to the YF17D vaccine (Fig. 7A). In contrast to the studies described above, we focused here not on the non-responders but on the positive “outliers” who mounted an “elite” response as defined above to YF17D (15/174). Elite vaccine responders tended to have lower alpha diversity (Fig. 7B), and the beta diversity significantly differed between the elite and non-elite groups (Fig. 7C, *p*=0.012). As shown in our first 3 cohorts, the MG revealed statistically significant metagenomic species contrasting “Elite” from the other subjects. Strikingly, the “Elite” subgroup was characterized by a clear relative overdominance of the genus *Enterocloster*, (including *E. citroniae, E. aldensis, E. lavalensis)* and *P. distasonis*, reportedly associated with gut dysbiosis, loss of MAdCAM-1 expression in ileal endothelial vessels, and blood recirculation of enterotropic regulatory and exhausted T cell subsets, all culminating in immunoresistance in patients with cancer (Fig. 7D)^42^ determining the TOPOSCORE for the “elite” individuals showed that 60% (9/15) of their scores were below the threshold of 0.79 (Grey+SIG1 categories), compared to 41.5% in the other subjects (Fig. 7E **left panel,** p=0.28), indicating that “elite” responders have a tendency towards immunosuppressive MGS. This was ascribed to a higher number of SIG1 bacteria (Fig. 7E **right panel,** p=0.18) and a lower number of SIG2 fecal bacteria (p=0.075) in Elite patients compared with the others. Moreover, correlating the toposcore classification with the immunological phenotypes of the YF17D cohort in peripheral mononuclear cells^51^ revealed higher percentages of circulating senescent CD4^+^CD57^+^CD127- Ki67^+^T cells, and lower percentages of effector CD8^+^ T-bet^+^ Tc1 cells in SIG1+ individuals at baseline (**Fig. S8A-B**).

Therefore, we conclude that the intestinal commensalism could at least partly account for the YF17D vaccine elite outliers, and propose the model that immunosuppressive MGS create an environment with reduced replicative control of the live vaccine leading to a higher antigen dose.

## Discussion

Here we show for the first time that failure to elicit Th1/Tc1 immune responses by different types of immunization protocols (either mRNA, nDC or long peptides) supposedly protective against cancer progression or severe SARS-CoV-2 infection as seen in 22–25 % of vaccinated individuals at the peak of the effector phase of cellular immunity correlates with an unhealthy microbiota. Hyporeactivity in cluster 0 individuals was not strictly vaccine antigen-specific and extended to other viral antigens. A healthy gut ecosystem may be defined based on the high prevalence of *Lachnospiraceae* (*Blautia* spp.*, Dorea* spp.*, Lacrimispora* spp.*, Lachnospira* spp) and *Oscillospiraceae* (*Faecalibacterium* spp., *Ruminococcus* spp.) family members and many more MGS associated with SIG2 in the TOPOSCORE^18,21,39^. This pattern of microbial ecosystem was significantly associated with cluster 1 and relatively absent in cluster 0 across the three independent cohorts. We extended this conclusion to humoral immune responses in the Welcome and EnDVR cohorts, although gut microbiota composition appeared to influence more the cellular than the humoral arm of immune responses to vaccines. Our study echoed several epidemiological studies conducted during the recent COVID-19 pandemic, analyzing the effects of ABX on seroconversion^54^. These authors draw associations between baseline and/or vaccination-induced changes in the taxonomic composition of stools with vaccine antigen-specific IgG titers^55–63^. However, the limitations of this study are the small effect size of cohorts, and the vast heterogeneity of subjects and vaccines that may weaken the main conclusion. Of note, this study does not bring causality links but only associations between gut microbiota composition and vaccine immunogenicity.

Interestingly, elite responders to a live attenuated immunizing formulation, such as the YF17D vaccine, exhibited a very peculiar gut taxonomic profile (characterized by a dominance of the genus *Enterocloster*), typically found in chronic inflammatory disorders^18^, ABX-treated and immunoresistant patients with cancer^21,39,42,64^. This apparently paradoxical result may be in line with the replicative potential of this live viral vaccine, that may be enhanced within the immunosuppressive metaorganism of “elite” subjects leading to a higher antigen dose and consecutive enhanced vaccine response. This argument is supported that increased circulating senescent proliferative T cells in the elite group and echoes the anticorrelation between basal levels of type 1 IFN surrogate markers (Cxcl10 and Siglec1 expressing myeloid cells) and vaccine immunogenicity observed by Santos-Peral et al.^51^ that might limit viral replication. It was known that live vaccines (like YF17D) differ in the signatures associated with stronger humoral responses compared to those described for other non-live vaccines that involve innate cells and typically include interferon stimulated genes and factors downstream of IRF7^65^. In addition, Santos-Peral et al.^51^ showed that the frequency of circulating activated (CD40L, Th1 cytokine+) and differentiated (CCR4+, CXCR3+, CCR6, and effector memory CCR7-CD45RA-)) CD4^+^ T cells at baseline served as a robust positive predictor of both humoral and cellular responses to the YF17D vaccine^51^. The specificities of these activated T cells are not known but might contain naturally occurring YF-reactive T cells, which can exist even without a previous flavivirus-antigen exposure, potentially leading to robust T cell responses following immunization^66^. Supporting this contention, Santos-Peral et al. unveiled that smoking, known to induce an active basal immunological status^67^ results in a more effective vaccine response to YF17D. Hence, we hypothesize that gut dysbiosis leading to the emergence of *Enterocloster* genus species could generate a proinflammatory status that increases the recirculation of exhausted and differentiated effector or regulatory T cells, that may share molecular mimicry with various antigens, as previously shown^42,64,68,69^.

Our findings highlighting the critical role of gut *Lachnospiraceae* and *Oscillospiraceae* in peripheral immune homeostasis suitable for optimal responses to inactivated vaccines (and to prevent overt responses to live vaccines) are also in line with pioneering experimental results. ABX uptake by pregnant dams or at the early stages of life impaired the immunizing capacity of vaccination, and germ-free animals failed to mount immune responses to candidate antigens unless orally fed with a complex microbiota^70–72^. The causal evidence for the impact of deliberate perturbations in the microbiome on the physiological states of humans has been brought up for the first time by Hagan and coll. who conducted an extensive multi-omics study in ABX-treated and control individuals vaccinated with the trivalent inactivated influenza vaccine^17^. The authors showed for the first time in subjects vaccinated with a H1N1-specific vaccine that a deviated taxonomic composition of the microbiota following ABX uptake can impair H1N1-specific neutralization by IgG1 and IgA antibodies^17^. These humoral immune perturbations correlated with the relative dominance and loss of *Enterobacteriaceae* and *Lachnospiraceae* family members, respectively, as well as with the depletion of secondary bile acids. These alterations triggered innate immune responses culminating in the activation of the inflammasome cascade in blood cells^17^. Toll-like receptor 5 (TLR5) was identified as the potential molecular link between ABX and defective humoral immunity since abrogation of TLR5-mediated sensing of flagellin impaired plasma cell and antibody responses to inactivated influenza vaccination in mice^73^. In fact, flagellin harbors adjuvant and antigenic features that can be recognized by TLR5, nucleotide-binding oligomerization domain-like receptor family CARD domain-containing protein 4, LY6/PLAUR domain containing 8 and cognate (Ig and TCR) receptors. A restricted repertoire of bacteria produces flagellins, mostly in the *Firmicutes* (such as *Lachnospiraceae* and *Oscillospiraceae* family members including *Roseburia* spp. and *Eubacterium* spp.) and Proteobacteria phyla^74^, that may contribute to the peripheral immune tonus of individuals. In early life, *Lachnospiraceae* are less prevalent within the microbiota, compared with *Bifidobacterium* spp. Fueling these observations, a correlative study between the taxonomic stool composition and the efficiency of oral and parenteral vaccines (polio virus, BCG, tetanus toxoid and hepatitis B virus) conducted in 48 Bangladeshi infants revealed positive and negative associations between the relative abundance of *Bifidobacterium longum subspecies infantis* versus *Proteobacteriales/Pseudomonadales* and IgG titers, T cell responses, as well as delayed-type hypersensitivity responses.

If the gut microbiome is essential for immune homeostasis and the host capacity to mount full-fledged immune responses to pathogenic insults^75–77^, pathological failures in turn compromise the gut barrier integrity and hence favor infections by opportunistic microbes^78,79^. Thus, extraintestinal cancers can promote a stress ileopathy, defined by degranulation of Paneth cells, accumulation of mucin producing goblet cells, ectopic proliferation of tyrosine hydroxylase expressing enteroendocrine cells and transient gut permeability^20^. This cancer-associated stress ileopathy depended on adrenergic receptor β2 signaling, paving the way to protracted dysbiosis with the expansion of *Enterocloster* spp. at the expense of other immunogenic *Lachnospiraceae* family members^20^, contributing to tumor progression. Similarly, Bernard-Raichon et al. showed in animal models that SARS-CoV-2 infection could induce gut dysbiosis associated with reactive Paneth and goblet cells, as well as with barrier permeability^80^. In COVID-19 patients, viral infection was associated with severe microbiota deviations characterized by reduced alpha diversity and the loss of anaerobic taxa^80^. Indeed, SARS-CoV-2 infection caused gut dysbiosis, either through gastrointestinal infection^81^ or through a systemic inflammatory response^82–84^. Opportunistic microbes from the dysbiotic gut microbiome may translocate into the bloodstream, a process that may be favored by virus-induced lymphopenia^85^ and antibiotic depletion of commensal gut bacteria^80^.

These accumulating lines of evidence suggest strategies for rendering vaccination campaigns more successful. First, the newly developed automated multiplexed T cell assays could complement tests that assess humoral (rather than cellular) immunity to identify nonresponders^86^. Second, user-friendly diagnostics tools of gut dysbiosis could help stratify individuals prone to be resistant to vaccines. Our recent attempts to fill this gap in patients with solid cancers still require prospective validation in a real-world scenario outside of the oncological ward^21^. Finally, interceptive measures reverting gut dysbiosis are being developed^41^ and may prove useful for reducing the risk of non-responses in population-based vaccination campaigns.

## Methods

### STUDY PARTICIPANT DETAILS.

The clinical cohorts included in this study complies with all relevant ethical regulations (Dutch Central Committee on Research Involving Human Subjects for MIND-DC, Comité de Protection des Personnes Est-II for UCPVax, WCM Institutional Review Board (IRB) for WELCOME, Comité de Protection des Personnes Sud-Ouest et Outre-mer III for COV3APHP and Comité de Protection des Personnes Ile-de-France XI for ONCOVID).

#### Patient cohorts and specimens.

Clinical trials and sample collection were performed in compliance with regulatory, ethical, and European General Data Protection Regulation and/or International Council for Harmonization E6(R2) guidelines for Good Clinical Practice requirements. The written informed consent was obtained for all patients in accordance with the World Medical Association’s Declaration of Helsinki as revised in 2013. We provide sex-related data (sex assigned at birth) and age (in years, from inclusion in protocol) for all study participants.

#### WELCOME. *Clinical Trial and Regulatory Approvals*.

The WELCOME (WEilL COrnell Medicine Employees) (protocol number IRB# 20–04021831) trial was conducted at Weill Cornell Medicine (WCM) and New York Presbyterian Hospital (NYP). The protocol is available at https://research.weill.cornell.edu/NYPWelcomeStudy. For details, refer to a previous report^87^.

#### COV3APHP. *Clinical Trial and Regulatory Approvals*.

bioMérieux S.A. is the promoter of the COV3APHP trial, which was approved by the local ethical committee (number ID-RCB: 2021-A00304–37). The trial was conducted at two French centers (Gustave Roussy and Cochin Institute). For details, refer to a previous report^25^.

#### ONCOVID. *Clinical Trial and Regulatory Approvals*.

The Gustave Roussy Cancer Center sponsored the ONCOVID trial. Protocol approval was obtained from an independent ethics committee (sponsor protocol number CSET 2020–3078, number ID-RCB: 2021-A01642–39, NCT04341207). The protocol is available at https://clinicaltrials.gov/ct2/show/NCT04341207. The trial was conducted at the French center Gustave Roussy. For details, refer to previous reports^25,85^.

#### MIND-DC. *Clinical Trial and Regulatory Approvals*.\

The MIND-DC (NCT02993315) trial is a double-blind, randomized, placebo-controlled phase III clinical trial in patients with localized melanoma (stages IIIB/C) with pre-planned stool sample collection, as previously described^22,23^. The protocol is available at https://www.clinicaltrials.gov/study/NCT02993315. Only patients included in the active treatment arm and with available MG data were included in this analysis (n=55).

#### UCPVax. *Clinical Trial and Regulatory Approvals*.

The Universal Cancer Peptide-based Vaccination (UCPVax) trial (NCT02818426) is a prospective multicenter phase I/II study in patients with metastatic NSCLC (stage IV). The protocol is available at https://www.clinicaltrials.gov/study/NCT02818426. For details, refer to previous reports^24^.

#### YF17D COHORT *Clinical Trial and Regulatory Approvals*.

174 healthy young adults, naïve to flavivirus infections and to Japanese encephalitis and yellow fever virus vaccine, were recruited at the Division of Infectious Diseases and Tropical Medicine (DIDTM) as well as the Department of Clinical Pharmacology, University Hospital, LMU Munich, Germany. Permission of the responsible institutional review board at the Medical Faculty of LMU had been granted prior to study initiation (IRB #86–16). Clinical cohort details are described in the ISRCTN registry, nr. 17974967^88^. Participants were recruited over five years (2015–2019), and all gave informed consent before receiving the subcutaneous YF17D vaccine (Stamaril; Sanofi Pasteur, Lyon, France). Samples (stools) were collected immediately before immunization and serum and PBMC additionally on day 28 post-vaccination.

#### EnDVR (Environmental Determinants of Vaccine Response) *Clinical Trial and Regulatory Approvals*.

The Environmental Determinants of Vaccine Response (EnDVR, NCT05239403) was an IRB approved (2020–1054), prospective, observational cohort study designed to elucidate the relationship between gut microbiome diversity, dietary patterns, and immune response to seasonal influenza vaccination, with secondary surveillance of SARS-CoV-2-specific immune markers. Between June 2021 and December 2024, the study enrolled healthy adult staff and student volunteers at MD Anderson Cancer Center, all of whom received the standard-of-care influenza vaccinations through the institution’s Employee Health program. SARS-CoV-2 vaccination status was self-reported. Eligibility criteria required participants to (i) be ≥18 years of age, (ii) possess an institutional medical record number, and (iii) consent to longitudinal biospecimen collection and survey completion; exclusion was limited to contraindications to vaccination. Repeat participation across annual seasons was permitted, although only a single enrollment per season was allowed.

Participants underwent a structured assessment schedule beginning up to four weeks prior to vaccination, including completion of dietary and health history questionnaires via REDCap (covering antibiotic/probiotic use, gastrointestinal symptoms, and prior vaccination), baseline venipuncture for hemagglutination inhibition (HAI) titer measurement, and initial stool sample collection for gut microbiome profiling. For surveillance of anti-SARS-CoV-2 titers, all enrollees self-reported height, weight, COVID-19 infection, and COVID-19 vaccination status. Post-vaccination follow-up involved serial blood draws at 4–6 and 8 weeks, paired with additional stool collections to evaluate longitudinal shifts in immune and microbial parameters. All blood samples were collected using standard venipuncture by trained phlebotomists and processed for vaccine-specific antibodies, flow and mass cytometry-based immune profiling, and SARS-CoV-2 serology, while stool samples were analyzed via metagenomic shotgun sequencing. Two parallel stool collection methods, ambient-stabilized (OMR-200 kit) and frozen were employed, and returned samples were banked at the Program for Innovative Microbiome and Translational Research (PRIME-TR) for downstream analyses. Only biospecimens collected within protocol-defined timepoints and free of processing deviations were included in the final dataset. All biospecimens were de-identified and tracked using unique study IDs via REDCap. Pilot analysis of the first 100 enrollees was conducted to validate sample integrity and optimize protocols. All biospecimens were de-identified using REDCap-linked study IDs, and data were securely stored on institutional firewalled servers. The primary immunological endpoint was defined as seroconversion, indicated by a ≥4-fold rise in post-vaccination HAI titers or an increase from baseline <1:10 to ≥1:40. Secondary endpoints included seroprotection rates (HAI titer ≥1:40) and associations with dietary and microbiome parameters.

## METHODOLOGY FOR IMMUNOMONITORING OF T OR B CELL RESPONSES.

### WELCOME cohort.

#### Isolation of PBMCs from fresh blood sampling.

30 mL venous blood samples were collected in sodium heparinized tubes (cat. #367874, BD). On the same day, blood was processed in a biosafety level 2 enhanced laboratory at Weill Cornell, New York, USA. PBMCs were freshly isolated by transferring blood into SepMate Tubes (cat. #85450, Stemcell Technologies) containing Ficoll Paque PLUS (cat. #17-1440-02, Cytiva) density gradient medium and centrifuging it using an Eppendorf 5910R centrifuge. After isolation, cells were washed twice, first using PBS (cat. #10010–031, Gibco) and then using RPMI 1640 (w/ L-glutamine) media (cat. #11875–093, Gibco).

After the second wash, cells were counted using Countess II Automated Cell Counter (Invitrogen) following manufacturer’s protocol and then aliquoted in cryovials (Thermo Nunc cat. #374512) containing 1mL of cryopreservation medium composed of 10% DMSO (cat. #D2650, Sigma-Aldrich) diluted in heat inactivated FBS (Gibco #10082–147). Immediately after adding freezing medium to cells, the cryovials were placed in a CoolCell FTS30 Cryopreservation Chamber (cat. #BCS-170, Biocision) and stored in a −80°C freezer. The samples were transferred from the −80°C freezer to liquid nitrogen the following morning (usually 16–24 hours after processing). If the cells were isolated on a Friday, they were transferred to liquid nitrogen on Monday morning.

#### Peptide pools.

Peptide pools spanning distinctive genomic sequences of the SARS-CoV-2 were designed. Full lists of the peptides contained in pools are provided in **Table S2A**. Peptides were synthesized by peptides & elephants GmbH (Berlin, Germany). All peptides were diluted in a stimulating solution provided by bioMérieux at 5μg/mL per peptide. This stimulating solution was used as a negative control (NIL) and a mitogen (MIT) i.e. phytohemagglutinin-P was used as a positive control.

The peptide pool PEPOrf encompassed distinct spike, membrane and nucleocapsid 15-mer sequences from the SARS-CoV-2 proteome overlapping by five residues. 18 immunodominant peptides were selected based on Tarke et al. and Nelde et al. works^89,90^.

The peptides from the WT (PEPwtRBD) or mutated (PEPmutRBD or PEPomRBD) were selected by dividing the 361–525 amino acid RBD sequence of the SARS-CoV-2 spike protein (RefSeq ID QHD43416.1) in nonoverlapping 15 amino acid segments. These peptide pools were composed of 11, 14 and 6 peptides respectively. For PEPmutRBD and PEPomRBD, variants emerged before Omicron mutations or Omicron (B.1.1.529) mutations were added to the sequences, respectively. The mutations from the variants were taken based on the information from https://outbreak.info/. For PEPmutRBD, we only considered mutations that were identified in at least 75% of the cases for a given strain. For PEPomRBD, we only select mutations that appear in around 90% of all publicly available sequences of the strains. The 19 peptides from the non-structural proteins’ pool (PEPnsp) were selected by dividing the sequence of two potential immunogenic regions of the SARS-CoV-2 non-structural proteins (based on *Swadling et al*. previous study^35^) in nonoverlapping 15 amino acid segments.

#### Antigen-specific PBMCs stimulation assay.

Frozen PBMCs were thawed, washed, and resuspended in RPMI 1640 glutamax media (cat. #61870036, GIBCO) supplemented with 1% penicillin/streptomycin (cat. #15140–122, GIBCO) and 10% decomplemented FBS standard (cat. #P30–3306, PAN Biotech) at a cell density of 0.2×10^6^ cells per mL. Viability and count were evaluated using a Vi-Cell XR Cell Counter (Beckman Coulter). PBMCs were plated in 96-well round-bottom TPP-treated culture plates (cat. #92097) (2×10^5^ PBMCs in 200μL) and stimulated with peptide pools (5μg/ml per peptide) for 72h at 37°C in 5% CO_2_.

#### Cytokine monitoring.

Supernatants from cultured cells from the PBMCs and whole-blood *(see* Antigen-specific whole-blood stimulation assay *section*) assays were monitored using Bio-Plex Pro Human Cytokine 27-plex Assays kit purchased from Bio-Rad (cat. #M500KCAF0Y) following the manufacturer’s protocol. Acquisitions and preliminary analyses were performed on the Bio-Plex^™^ 200 system (serial no. X10020093421) and Bio-Plex Manager^™^ (version 6.2) software, both purchased from Bio-Rad.

#### Positivity threshold determination for cytokine concentration using multiplex assays.

For multiplex assays, a five-parameter logistic regression was fitted for each cytokine based on the Allophycocyanin (APC) mean fluorescent intensity of standard dilution samples using Bio-Plex Manager^™^ (version 6.2) software (Bio-Rad). This model was then used to calculate the concentration of each sample of unknown concentration. A ratio was computed for each cytokine using the cytokine concentration measured in response to each peptide pool (PEPOrf, PEPwtRBD, PEPmutRBD, PEPomRBD, PEPnsp) divided by concentration of their biological controls (NIL). A delta was also computed using the concentration measured in response to each peptide pool and their respective control. For PBMC stimulation, positivity was defined as a delta superior to the absolute value of the smallest delta computed divided by two, a ratio superior to two and a delta between the sample pre-vaccination and the sample post-vaccination of the same patient superior to zero.

#### IgG anti-RBD-S1 dosage.

Vidas SARS-CoV-2 IgG (ref 423834) (bioMérieux, France) is an automated qualitative CE-*in vitro* diagnostic (IVD) assays developed for the Vidas family of instruments and based on a two-step enzyme immunoassay combined with an enzyme-linked fluorescent assay (ELFA) detection technique. A solid-phase receptacle coated with the antigen (recombinant SARS-CoV-2 receptor-binding domain [RBD] of the viral spike protein) serves as both solid-phase and pipetting device. After the sample dilution step, SARS-CoV-2-specific IgG are captured on the coated antigen, and unbound components are washed out. In the second step, human IgG are specifically detected by mouse monoclonal antibodies conjugated to alkaline phosphatase and directed against human IgG. The test is interpreted as negative when *i* < 1.00 and positive when *i* ≥ 1.00. The positivity cutoff values for IgG tests were determined from a healthy prepandemic cohort (120 samples collected prior to August 2019).

### COV3APHP and ONCOVID cohorts.

#### Fresh blood sampling.

Whole human venous peripheral blood samples (10–30mL) were collected in heparinized tubes, BD Vacutainer LH 170 U.I., from Dutscher (cat. #367526).

#### Antigen-specific whole-blood stimulation assay (COVID IGRA bioMérieux Assay).

Fresh blood (300μL) collected in heparinized tubes was plated in low binding 48-well flat-bottom with low evaporation lid plate (cat. #353078, Dutscher) and stimulated for 22 hours at 37°C under 5% CO_2_ with peptide pools (300μL) described in Peptide pools *section* above, resulting in a 2-fold dilution. Following incubation, supernatant was collected by a 460g, 10min centrifugation using a 5810R centrifuge from Eppendorf and stored in SafeSeal microcentrifuge Low binding polymer technology tubes (cat. #27200, BioScience, Inc. Sorenson) at −20°C.

#### Cytokine monitoring and positivity threshold.

The concentration of IFNγ in the supernatant was measured using the VIDAS^®^-30 semi-automated platform (serial no. IVD 3004335) and IFNγ RUO protocol from bioMérieux. The positivity range was from 0.08 to 8 IU/mL. Positivity thresholds were defined at 0.08 IU/mL. The IFNγ response was defined as positive when the IFNγ concentration of the test was above threshold and the negative control was below threshold or when the IFNγ concentration of the test minus the IFNγ concentration of the negative control was above threshold. All positive controls were >8 IU/mL.

### MIND-DC cohort.

DTH responses were performed as previously reported^22^ after 3 priming vaccinations. Production of IFNγ, IL-2, IL-5, and IL-10 was measured in the supernatants by cytometric bead array according to the manufacturer’s instruction (eBioscience, Vienna, Austria or BD Biosciences, San Jose, CA) after 24 hours of co-culture.

### UCPVax cohort.

hTERT-specific CD4 T cell responses were assessed in peripheral blood using IFNγ ELISPOT at baseline, day 29 (after 6 priming vaccinations), prior each boost vaccination and at the end of treatment visit as previously described^24^. Only ELISPOT results after 6 priming vaccinations were used in this study.

### YF17D cohort.

As described in detail in Santos-Peral et al.^51^, the neutralizing antibody titer at day 28 post vaccination was quantified by a Fluorescence Reduction Neutralization Test (FluoRNT), and the frequency and functionality of YF17D-specific CD4 and CD8 T cells was measured by intracellular cytokine staining of PBMC samples re-stimulated ex vivo with the YF17D virus using an extended 14-color flow cytometry panel covering both the CD4 and CD8 T cell response. The functional score of the antigen-specific T cell response was estimated using COMPASS analysis^52^. Hybrid hierarchical k-means clustering from the R factoextra package was used to classify the vaccinees into good/elite, average and poor responders separately for CD8 T cell readouts, CD4 T cell readouts and the neutralizing antibody response.

### EnDVR cohort.

Quantification of SARS-CoV-2 spike antibodies in serum was performed following a protocol established and kindly shared by Dr. Florian Kramer^49^. Briefly, heat-inactivated serum samples were serially diluted in 1% (w/v) non-fat dry milk (NFDM) prepared in 0.05% PBS-T (PBS with 0.05% Tween-20) through a four-step dilution series (1:5, 1:50, 1:400, and 1:3200) in triplicate wells. Coated plates were washed three times with 0.05% PBS-T and then blocked for 1 hour at room temperature with 200 μL of 3% (w/v) NFDM per well. Each well was loaded in triplicate with 100 μL of 1:3200 diluted patient serum, positive control serum, or negative control serum. A ten-point standard curve dilution was generated using rabbit anti-RBD monoclonal antibody at 1 μg/mL working stock serially diluted in 1% NFDM from 50,000 pg/mL to 100 pg/mL). Plates were incubated for 2 hours at room temperature with slight, periodic shaking. Following primary incubation, wells were washed three times with 0.5% PBS-T and then loaded with 50 μL of horseradish peroxidase (HRP)-conjugated secondary antibodies: anti-human IgG-HRP (1:375 dilution in 1% NFDM) for patient and control serum wells, and anti-rabbit IgG-HRP (1:375 dilution in 1% NFDM) for standard curve dilution wells. Plates were incubated at room temperature for 1 hour and then washed three times with 0.5% PBS-T. Colorimetric signal detection was performed using freshly prepared o-phenylenediamine dihydrochloride (OPD) substrate solution. Optical density was immediately recorded at 490 nm using a calibrated microplate reader. Final concentrations were expressed in pg/mL, adjusted for blank signal, and normalized across technical replicates.

## HLA GENOTYPING.

### Isolation of PBMCs from fresh blood sampling.

On the same day blood was collected, part of it was transferred in Leucosep-50mL tubes purchased from Greiner Bio-One (cat. #227290). PBMCs were freshly isolated by the density gradient centrifugation lymphocyte separation medium (cat. #CMSMSL01–01, Eurobio Scientific) according to the manufacturer’s instructions (Leucosep tubes, Greiner; Biocoll, Bio&SELL) using a MF48-R centrifuge from AWEL Industries (cat. #20023001). PBMCs were collected in a centrifuge tube, 50mL, TPP from Dutscher (cat. #91050), washed with phosphate-buffered saline (PBS) 1X solution, resuspended in 1mL of cryopreservation medium (CryoStor CS10 purchased from STEMCELL Technologies (cat. #5100–0001)), and transferred in CryoTube vials from Thermo Fisher Scientific (cat. #377267) (two cryovials per patient). Cryovials were finally conserved for 24 hours at −80°C in a cryo-freezing container (Mr. Frosty, Thermo Fisher Scientific) before storage in liquid nitrogen.

### DNA extraction.

DNA was extracted using 200μL of blood, with the EZ1 DNA Blood 200μl kit from QIAGEN and the EZ1 instrument (QIAGEN) according to the supplier’s recommendations. The quality and quantity of the extracted DNA were studied on Fragment Analyzer (Agilent technology).

### HLA sequencing.

High-resolution HLA genotyping was performed on a next-generation sequencer (MiSeq Illumina) employing the NGSgo^®^-MX11–3 kit (GenDx), following the manufacturer’s protocol. Briefly, the protocol involved locus-specific amplification of the complete HLA region in 3 pools (30ng of the isolated DNA): Pool A targeting HLA-A (3.1kb), HLA-DRB1 (7.5kb), HLADPB1 (5kb), HLA-DRB3 (7.5kb), HLA-DQA1 (5.8kb); Pool B targeting HLA-B (3.4kb), HLA-DQB1 (6.7kb), HLA-DRB5 (7.4kb) and Pool C targeting HLA-C (3.4kb), HLA DPB1 (5.7kb), HLA-DRB4 (4.3kb), HLA-DPA1 (5.5kb). Generated sequences were assessed for quality, sequenced and the alleles were interpreted using NGSengine^®^ software (GenDx).

## METAGENOMICS ANALYSIS.

### Stool collection.

WELCOME trial participants put no more than 75mg of solid stool into the sterile 76×20mm feces tube (cat. #80.734.001, SARSTEDT). The stool containers were directly frozen at −80°C. MIND-DC trial’s fecal samples were collected at baseline (prior to vaccination) and after first cycle as per-protocol^22^. UCPVax trial fecal samples were collected at baseline (prior to vaccination) and at the end of treatment visit as per-protocol^24^. YF17D cohort’s fecal samples were collected at baseline (prior to vaccination with the YF17D vaccine) and frozen at −80°C.

### DNA extraction and shotgun metagenomics sequencing.

Overall, 197 fecal samples from 153 patients were sequenced with whole genome sequencing technology and analyzed. DNA was extracted from aliquots of fecal samples using the DNeasy PowerSoil Pro Kit (Qiagen) following the manufacturer’s instructions. Sequencing libraries were prepared using the Illumina^®^ DNA Prep, (M) Tagmentation kit (Illumina), following the manufacturer’s guidelines. A cleaning step on the pool with 0.7x Agencourt AMPure XP beads was implemented. Sequencing was performed on a NovaSeq 6000 S4 flow cell (Illumina) at the internal sequencing facility at University of Trento, Trento, Italy. Raw sequenced reads were QCed using the pipeline available at https://github.com/SegataLab/preprocessing. Briefly, low-quality reads (Q<20), short reads (<75bp), and reads with at least 2 ambiguous bases were discarded. Then, host DNA contaminants were removed (hg19 and phiX174 Illumina spike-in). We then obtained an average of 50 million reads per sample in the MIND-DC cohort, 72.8 million reads per sample in the UCPVAX trial, and 77.4 million reads on average per sample in WELCOME. Twelve samples did not pass internal control and were excluded from the analysis. For each metagenome we profiled the taxonomic and functional potential compositions with MetaPhlAn 4 (database vJan21)^36^, which is based on the SGB system^37^ and HUMAnN 3.8^91^, respectively.

### Metagenomics methods for the EnDVR trial samples.

Genomic DNA was extracted from stool samples using the QIAamp Fast DNA Stool Mini Kit (Qiagen), with a modification that added an extra bead-beating lysis step. Each tube, containing the stool sample, was supplemented with a 3.2-mm steel bead, approximately 150 mg of zirconium beads, and lysis buffer. The samples were bead-beaten for a total of 8 minutes at 3,800 rpm (BioSpec) to ensure bacterial lysis for DNA extraction, following the manufacturer’s protocol. Fecal microbial DNA was then processed into individual libraries using the Illumina DNA Prep Kit (Illumina), pooled, and loaded onto the Illumina NovaSeq 6000 v1.5 Reagent Kit on the NovaSeq 6000 platform (Illumina) for sequencing. Sequencing was conducted following the 2×150 bp paired-end read protocol, according to the manufacturer’s instructions.

## STATISTICAL ANALYSIS.

All calculations, statistical tests, and data visualization were performed using R v4.1.2 or Python 3. All analyses were performed on independent samples. Group comparisons were performed using the nonparametric Wilcoxon–Mann–Whitney test with the wilcox.test R function or the Student t-test in scipy python package (ver. 1.11.4). The comparison of categorical data was performed using the Fisher’s exact test or Chi-squared test with the fisher.test or chisq.test R functions and the chi.contingency test in scipy python package. Unsupervised hierarchical clustering was performed with the R-package hclust, using the binary distance and ward.D2 clustering method. All hypothesis tests were two-sided and considered statistically significant when p-value<0.05.When appropriate, p-values were adjusted for multiple testing with the False Discovery Rate method implemented in the R p.adjust function. Graphical illustrations were drawn using the standard R packages dedicated to the data visualization (ggplot2, ggpubr, pheatmap, circlize, ggVennDiagram, cowplot and gtsummary).

### HLA analysis.

For HLA analysis, the two cohorts used were divided into two different groups based on their PEPwtRBD IFNγ longitudinal reactivity. Negative group was composed of participants with a longitudinally full negative PEPwtRBD IFNγ reactivity and positive group was composed of participants with at least one positive PEPwtRBD IFNγ sample longitudinally. Comparison of allele frequencies between both groups was carried out using a logistic regression model using the midas HLA package and all statistical values were considered significant at *p*<0.05.

### Metagenomics analysis.

Calculations of alpha-diversity were done in Python: alpha-richness was computed as the count of positive SGBs in each sample profile; alpha diversity was computed as natural log Shannon entropy of relative abundances corresponding proportions and as inverse-Simpson of the proportions. Alpha-diversity and richness differences in immune activation clusters were evaluated with Student t-test. Beta-diversity analyses were conducted using pairwise Bray-Curtis dissimilarities considering only baseline sample MetaPhlAn 4 profiles. Dimensionality reduction was conducted via multidimensional scaling (MDS) performed on the dissimilarity matrices of each cohort based on the full SGBs profiles, and between-cluster differences were evaluated using PERmutational Multivariate ANalysis Of VAriance (PERMANOVA) as implemented in the scikit-bio package in Python (ver. 0.5.8). Inter- vs. intra-individual beta-diversity analysis was conducted by subsampling dissimilarity matrices of the WELCOME and UCPVax cohorts including baseline and 22 and 10, respectively, follow-up samples.

### Non-linear regression and meta-analysis approaches on microbial features.

In order to assess differentially abundant microbiome SGB relative abundances in either cluster 1 (immune activated) or cluster 0 (non-reactive), we implemented a regression model based on gamma-distributed residuals with log-link (log-gamma regression) which is intrinsically related to the microbial SGBs’ relative abundances distributions. Specifically, cluster 0 and 1 were encoded as categorical variables and the one model was fit for each microbial SGBs relative abundances array in each cohort independently including in the model sex and age in the WELCOME, MIND-DC and UCPVax cohorts. Models were implemented in a custom python script using the statsmodels library (ver. 0.14.0). The coefficient from the models relative to the immune activation cluster was taken as an effect-size measure with its associated p-value. FDR adjustment was performed via Benjamini-Yekutieli procedure on the distribution of all the p-values from all the SGBs tested in each cohort. Meta-analysis was performed for all SGBs that were found in a minimum of two cohorts pooling together coefficients from the aforementioned models and synthesizing them using inverse-variance weighting implemented in a custom python script. In brief, log-gamma models coefficients were pooled together with their standard error estimations and a random-effect meta-analysis was computed using the Paule-Mandel heterogeneity estimation. Meta-analysis p-values from all SGBs were adjusted for FDR via Benjamini-Yekutieli and the significance threshold was set to q-value<0.2. Microbial genes differential abundance analysis was performed on the read-per-kilobases (RPK) estimated by HUMAnN 3.6 grouped according to the Enzyme Commission (EC) number ontology (using the *‘humann_renorm’* script). As per count-based data, we implemented a similar script based on negative binomial regression models for discrete data available in the *statsmodels* library. Coefficients from these models were pooled next in meta-analysis as before. Due to the larger number of features tested, significance thresholds were set to q-value<0.1 for single-cohort analyses and q-value<0.01 and <0.1 for meta-analysis.

### Machine learning approaches.

We used MetAML^92^ to build RF models (based on the scikit-learn implementation in Python, ver. 1.2.2) to predict the experimental readout of the patients’ immune activation clusters by the SGB-level microbiome composition estimated by MetaPhlAn 4. Specifically, we performed 10-times iterated, 10-fold stratified cross-validations on the WELCOME and the MIND-DC cohorts independently; UCPVax was evaluated through 10-times iterated, 5-fold cross-validations, to compensate for the reduced number of samples. Our models entailed 1,000 estimator trees, 10% of the total feature space into each tree, no-fixed maximum depth, a minimum of 3 samples per leaf, and Gini as impurity criterion. Performances were tested therefore across a total of 100 models in the WELCOME and MIND-DC cohorts and 50 models in the UCPVax cohort, and were evaluated using the area under the ROC curve. We then took advantage of the implementation of MetAML which permits to average the feature rankings from training sets only, preserving the information of the corresponding test sets and assessing their performance. We next performed three similar experiments based on 10-times iterated, 10- and 5-fold cross-validations, in which, for each cohort iteratively, the other two cohorts were added to each training phase of the algorithm (i.e. a hybrid approach in which a cross-validation is mixed with a leave-one-dataset-out (LODO), termed X-LODO). We next built the cross-prediction matrices for all the kind of feature sets used: microbiome only, microbiome plus sex, age, and cancer status, and sex, age, and cancer status, plus the two tests involving microbiome features performed on the top-40 features as selected by only considering the training folds to avoid overfitting. The cross-prediction (presented in **Fig. S4**) is a matrix in which each slot correspond to the result of a machine-learning algorithm trained on the cohort reported on the corresponding row and tested on the cohort reported on the corresponding column (i.e. which recapitulates all possible combinations of transfer of information from one cohort to another), and in which the diagonal accommodates the cross-validations. To these matrices we added the LODO and the X-LODO rows.

## Supplementary Files

This is a list of supplementary files associated with this preprint. Click to download.

- Supplementaltable4OCT52025.pdf
- Supplementaltable5a.2OCT52025.pdf
- FigureS3exS5JAN19.pdf
- Supplementaltable6NOV52024.pdf
- FigureS1JAN19.pdf
- Supplementaltable2BNOV52024.pdf
- FigureS2JAN19.pdf
- Supplementaltable8OCT52025.pdf
- Supplementaltable3NOV52024.pdf
- Supplementaltable5bOCT52025.pdf
- Supplementaltable2ANOV52024.pdf
- Supplementaltable1NOV52024.pdf
- FigureS52k25SEPT21.pdf
- FigureS4exS3JAN19.pdf
- Supplementaltable5dOCT52025.pdf
- FigureS6JAN19.pdf
- Supplementaltable7NOV52024.pdf
- Supplementaltable5a.1OCT52025.pdf
- FigureS72k25SEPT22.pdf
- Supplementaltable5cOCT52025.pdf
- FigureS82k25SEPT21.pdf

**Figure S1. HLA genotypes and COVID-19 vaccine failures to mount RBDwt-specific Th1 responses. A.** Graphical representation of the two patient cohorts that underwent HLA phenotyping. Discovery cohort is composed of COV3APHP (French HCW) and ONCOVID (cancer) patients ***(upper panels)*** (refer to **Table S1A**) and validation cohort is composed of only COV3APHP patients ***(lower panels)*** (refer to **Table S1B**). **B.** Longitudinal PEPwtRBD-specific IFNγ concentration (y axis) after whole-blood stimulation for 22h and IFNγ quantification on VIDAS^®^-30 for each sample of each patient of both discovery and validation cohorts (n=173). Each dot represents the log10-transformed concentration of IFNγ of one sample. **C.** Percentage and number of patients positive (black) or negative (grey) for the vaccine/SARS-CoV-2 virusinduced PEPwtRBD-specific T cell responses within both discovery and validation cohorts (n=173) overtime. The discovery cohort COV3APHP + ONCOVID was composed of 59 individuals who were tested once (n=33) or twice (n=19) or three times (n=6) or four times (n=1). Out of 59, 17 (29%) were negative and 42 were positive at least once. The validation cohort COV3APHP was composed of 114 individuals who were tested once (n=5) or twice (n=21) or three times (n=17) or four times (n=70) or five times (n=1). Out of 114, 28 (25%) were negative and 86 were positive at least once. **D.** Intersection between the T cell reactivities against WT versus mutated peptides of the S1-RBD sequence. Barplots representing the percentage and number of patients within discovery and validation cohorts (n=173) that are longitudinally PEPwtRBD-IFNγ positive/negative and/or PEPmutRBD-IFNγ positive/negative (positive in black, negative in grey) after whole-blood stimulation for 22h and IFNγ quantification on VIDAS^®^-30. Fisher’s exact test was used to compare the number of positive patients in both groups. **E-F.** Volcano plots of the discovery cohort (n=59) (E) and the validation cohort (n=114) (F) were generated performing a logistic regression analysis of the contribution of each HLA allele to differentiate PEPwtRBD-IFNγ longitudinally negative vs positive (as shown in **Fig. S1B-C**). The coefficient regression was computed on the x axis while the −log10 of the p-values deriving from the logistic regression are represented on the y axis. Statistically significant alleles (*p*<0.05) are annotated above dashed line which represents *p*=0.05.

**Figure S2. Non-reactivity to other structural and non-structural SARS-CoV-2 epitopes and correlations with RBDwt-related non-reactivity in WELCOME. A-E.** Left panels depict bicolor map of cytokine secretion in a non-supervised hierarchical clustering of HCW after 72h PBMC stimulation with PEPmutRBD peptide pool (A, n=74 HCW), with PEPomRBD peptide pool (B, n=75 HCW), with PEPOrf peptide pool (C, n=75 HCW), with T cell mitogen (MIT= phytohemagglutinin-P) (D, n=74 HCW), with PEPnsp peptide pool (E, n=75 HCW) (positive in black, negative in grey). Patients were ordered in columns and cytokines were ordered in rows by unsupervised hierarchical clustering using ward.D2 clustering method and binary distance calculation method. Cytokines selected for the clustering were those from Fig. 1B. Heatmaps were cut in 2 clusters of patients (purple: nonreactive (cluster 0), blue: polyreactive (cluster 1)). Right panels depict barplots representing the percentage and number of patients that are PEPwtRBD-reactive/non-reactive vis-à-vis of the other reactivities (purple: non-reactive (cluster 0), blue: polyreactive (cluster 1), *see left panels*). Fisher’s exact test was used to compare the number of cytokine-positive patients across clusters.

**Figure S3. Top-40 features variable importance per cohort using the random forest (RF) machine learning integrating all cohorts in the algorithm. A.** Top-40 ranked species from a RF algorithm to distinguish cluster 1 from cluster 0 of immune activation in the WELCOME cohort as defined with PEPOrf (**Fig. S2C**). Algorithm was trained and tested on the WELCOME cohort via a 10-fold, 10-times iterated cross-validation to which the other two cohorts were added as training boosters. Species have been extracted considering only the training folds to avoid overfitting, and colored by the immune activation cluster (orange for cluster 0 and blue for cluster 1) based on the higher group mean relative abundance. Effect-size are taken from a log-gamma regression on the cluster of immune activation adjusted by sex and age, colored by direction of the effect-size, and hatching indicates the *q*<0.2. Prevalence and relative abundances are reported by the patients’ group. **B.** Same as A, extracting the forty species from an algorithm attempting to distinguish clusters of immune activation in the WELCOME cohort as defined by PEPwtRBD (Fig. 1B). **C.** Same as A, extracting the forty species from an algorithm attempting to distinguish clusters of immune activation in the MIND-DC cohort (clusters are defined in Fig. 3B). **D.** Same as A, extracting the forty species from an algorithm attempting to distinguish clusters of immune activation in the UCPVax cohort (clusters are defined in Fig. 4B).

**Figure S4. Prediction of response using MGS signatures and solely age and sex.** Random forest cross prediction matrices with different feature sets and assessment strategies - from left to right, random forest cross prediction matrix of the three cohorts using different feature sets: microbiome only; top-40 species as ranked by the random forest algorithm internally; microbiome, sex, age, and cancer status; top-40 predictive feature among microbiome, sex, age, and cancer status; sex, age, and cancer status only. Each slot in the top matrix represents the AUC of an algorithm trained on the cohort written on the corresponding row and tested on the cohort reported on the corresponding column. Slots on the matrix diagonal represent ten times repeated, tenfold cross validations (CV). Bottom rows report, for each cohort, the Leave-one-dataset-out (LODO), and the X-LODO, where for each cohort a CV is performed supplementing the training folds with the other two cohorts.

**Figure S5. COVID-19 vaccine and viral infections induce broader Th1 immune responses that can eventually be protective against the Omicron BA.1 outbreak. A.** Graphical representation of the COV3APHP patient cohort’s protocol with fecal and blood collection and timelines between vaccination and sampling (refer to **Table S6**). **B.** Bicolor map of cytokine secretion in a non-supervised hierarchical clustering of HCW after 22h whole-blood stimulation with PEPOrf peptide pool (positive in black, negative in grey). Patients (n=91) were ordered in columns and cytokines were ordered in rows by unsupervised hierarchical clustering using ward.D2 clustering method and binary distance calculation method. Cytokines with less than 10% frequency were excluded of this clustering. Heatmap was cut in 2 clusters of patients (green: cluster α and purple: cluster β). Future breakthrough COVID-19 infection events (post blood drawing and vaccine) are indicated in the first line. Positive threshold was defined as a fold ratio of the cytokine concentrations for PEPOrf over its negative control superior to 2 for each cytokine. **C.** Volcano plot depicting the most significant cytokines after whole-blood stimulation with the PEPOrf peptide pool between cluster α (green) and cluster β (purple) of panel B. Volcano plot was generated computing delta of the percentages of positive patients in cluster α and the percentages of positive patients in cluster β for each cytokine (x axis) with the −log10 of the p-values deriving from Fisher’s exact test for each cytokine. **D.** Left panel depicts Barplots representing the percentage and number of patients who will experience a future COVID-19 breakthrough infection (post blood drawing and vaccine) in each cluster of the panel B (green, n=13 and purple, n=78). Idem for right panel but considering reactivity to PEPwtRBD. Fisher’s exact tests were used to compare the number of future COVID-19 infected-patients in the clusters. **E.** Left panel depicts bicolor map of cytokine secretion in a non-supervised hierarchical clustering of HCW after 22h whole-blood stimulation with PEPomRBD peptide pool (positive in black, negative in grey). HCW (n=79) were ordered in columns and cytokines were ordered in rows by unsupervised hierarchical clustering using ward.D2 clustering method and binary distance calculation method. Cytokines with less than 10% frequency were excluded of this clustering. Heatmap was cut in 2 clusters of patients (orange: non-reactive (cluster ε) and dark-blue: reactive (cluster λ)). Past SARS-CoV-2Omicron BA.1 infections are indicated in the first line. Positive threshold was defined as a fold ratio of the cytokine concentrations for PEPomRBD over its negative control superior to 2 for each cytokine. Right panel depicts barplots representing the percentages and number of patients experiencing SARS-CoV-2-Omicron BA.1 infections within cluster ε and λ HCW and Fisher’s exact test.

**Figure S6. *Faecalibacterium prausnitzii* SGB15316 is clinically significant in two independent datasets of cancer patients. A-B.** The Kaplan-Meier survival curves based on the prevalence of a different *F. prausnitzii* species-level genome bins (SGBs) (presence in green, absence in black) for overall survival (OS) in a cohort of 499 advanced NSCLC patients treated in 1st line or further with immunotherapy-based regiments (reported in^21^) (A) or relapse-free survival (RFS) in patients with stage III melanoma from the MIND-DC trial (reported in^23^) (B). The p-value corresponds to the comparison of the Kaplan-Meier curves using the log-rank test.

**Figure S7. Impact of the gut microbial composition on the rise of RBD spike -specific antibody titers after the 3**^**rd**^ **recall SARS-CoV2 vaccine in the EnDVR cohort. A.** Graphical timelines of the immunization protocol against SARS-CoV2 spike protein with the stool and serum harvesting (Table S8). **B.** Taxonomic alpha-diversity of HV samples was estimated using the observed taxa (richness). Alpha-diversity metrics were computed for nonresponder (cluster 0) and responder (cluster 1) vaccinees (left panel). Responders were defined by a >2 fold increase in Ab titers post booster, with a titer >20ug/ml post-booster. Betadiversities (principal coordinate analysis, PCoA) of fecal microbiota (microbial relative abundances) according to the clustering (right panel). **C.** Supervised analysis using partial least square discriminant analysis (PLS-DA) and variable importance plot (VIP) to identify the most discriminant microbial species among non-responders (cluster 0) and responders (cluster 1). ANOSIM and PERMANOVA define the separation of the groups; p values define the significance of group separation after 999 permutations of the samples. Mann-Whitney U test p values (*p<0.05, **p < 0.01, ***p < 0.001) are indicated. **D**. Spearman correlations between the S score (SIG2/SIG1) and the difference in the antibody titers before and after the booster in cluster 0 and cluster 1 vaccinees. **E.** Toposcore using the two or three categories and Fischer exact test or Chi-square test p values, between cluster 1 and cluster 0.

**Figure S8. Immune phenotypes associated with SIG1+ YF vaccinees. A.** Significant immunomics (flow cytometric analysis)-based parameters separating SIG2+ from SIG1+ vaccinees at baseline before YF vaccination. The XGBoost binary classification model with 40 immune parameters adjusted on age+sex, the Boruta selection for best features, and a SHapley Additive exPlanations values for impact output of each parameter were used. Selected features are ranked by importance in the SIG1+ vs SIG2+ status. SHapley Additive exPlanations values for impact output of each parameter are represented for each vaccinee. Each dot represents one individual and the color represents the measured continuous variable for each feature. Negative SHAP values are associated with SIG2+. Conversely, positive SHAP values are associated with SIG1+. The feature is marked in purple when SIG1 or SIG2 risk increases with its value while marked in orange in the opposite sense. **B-C.** Box plots illustrate the raw values of the features (absolute numbers /mm3 WBC (GL-Day0) or percentages (Freqof) of circulating CD4+ or CD8+ T cells) retained by the model in the two groups SIG1+(black dots) vs SIG2+ (green dots) with Mann- Whitney tests with FDR adjusted p values.

Table S1. Characteristics of the individuals assessed for HLA genotypes and RBD-S1-specific Th1 responses.

Table S1a. PEPwtRBD reactivities in the COV3APHP and ONCOVID cohorts studied for HLA allele phenotypes (n=59).

Table S1b. PEPwtRBD reactivities in the COV3APHP cohort studied for HLA allele phenotypes (n=114).

Table S2. Peptide lists and their HLA binding in silico prediction.

Table S2a. List of amino acid residues spanning distinct structural and non-structural proteins.

Table S2b. MHC class I and II binding predictions of the peptide list (S2A).

Table S2b.1. In silico binding capacities of S1-RBD peptides to MHC class I molecules.

Table S2b.2. In silico binding capacities of S1-RBD peptides to MHC class II molecules.

Table S3. HCW and cancer patient characteristics according to immune responsiveness.

Table S3a. HCW and cancer patient characteristics in each cellular responses cluster 0 versus 1.

Table S3b. HCW characteristics in each humoral responses cluster Low versus High in the WELCOME cohort.

Table S4. Raw data of cytokine release per peptide pool in WELCOME.

Table S5. Significant metagenomics species retained in the MetAML model according to the peptide pool or the cohort and in the meta-analysis.

Table S5a.1. WELCOME cohort for PEPwtRBD.

Table S5a.2. WELCOME cohort for PEPOrf.

Table S5b. MIND-DC cohort.

Table S5c. UCPVax cohort.

Table S5d. Meta-analysis.

Table S6. COV3APHP HCW characteristics.

Table S7. Details of the IFNγ ELIspot assay of UCPVax.

Table S8. EnDVR cohort of US healthy volunteers drawn before and after the booster of the SARS-CoV2 vaccine.

## Data Availability

Quality-controlled metagenomic sequences for the WELCOME, UCPVax, and MIND-DC are available in the European Nucleotide Archive (ENA) under the accession numbers PRJEB75003, PRJEB75004, and PRJEB66197, respectively. Other data that support the findings of this study are available from the corresponding author upon reasonable request.

## Abbreviations

Ab: Antibody
ABX: Antibiotics
AUC: Area Under the Curve
avg.: average
BCG: Bacille de Calmette Guerin
coeff.: coefficient
CoV-2-HCoV: Coronavirus 2 Human Coronavirus
COVID-19: Coronavirus disease 2019
CTLA-4: Cytotoxic T-lymphocyte associated protein 4
DC: Dendritic Cells
DTH: Delayed-type hypersensitivity
DTP: Diphtheria-TetanusPertussis
EC: Enzyme Commission
ELFA: Enzyme Linked Fluorescent Assay
ELISpot: Enzyme-linked immunosorbent spot
HCW: Health Care Workers
HIV: Human Immunodeficiency Virus
HLA: Human Leukocyte Antigens
hTERT: human telomerase reverse transcriptase
IFNγ: Interferon gamma
Ig: Immunoglobulin
IL: Interleukin
MetaPhlAn 4: Metagenomic Phylogenetic Analysis
MG: Shotgun Metagenomics Sequencing
MGS: Metagenomics Species
MHC: Major Histocompatibility Complex
nDC: natural Dendritic Cells
NSCLC: Non-Small-Cell Lung Cancer
PBMC: Peripheral Blood Mononuclear Cells
PERMANOVA: PERmutational ANalysis Of Variance
PD-1: Programmed cell Death protein 1
PD-L1: Programmed death-ligand 1
PV: Polio Virus
RBD: Receptor Binding Domain
RF: Random Forest
ROC: Receiver Operating Characteristic
RUO: Research Use Only
S1: Spike-1
SARS-CoV-2: Severe Acute Respiratory Syndrome Coronavirus 2
SGB: Species-level Genome Bin
spp.: species
TAA: Tumour Associated Antigen
TLR5: Toll-like receptor 5
TT: Tetanus Toxoid
UCPVax: Universal cancer peptide-based vaccine
YF: yellow fever
YF17D: yellow fever 17D vaccine strain
VOC: Variants Of Concern
WT: Wild Type
